# *Ganoderma adspersum* (Ganodermataceae): Investigation of Its Secondary Metabolites and the Antioxidant, Antimicrobial, and Cytotoxic Potential of Its Extracts

**DOI:** 10.3390/ijms25010516

**Published:** 2023-12-30

**Authors:** Raichan Chafouz, Sofia Karavergou, Olga St. Tsiftsoglou, Pavle Maskovic, Diamanto Lazari

**Affiliations:** 1 Laboratory of Pharmacognosy, Faculty of Health Sciences, School of Pharmacy, Aristotle University of Thessaloniki, 54124 Thessaloniki, Greece; reichan.chfz@gmail.com (R.C.); sofiakaravergou@gmail.com (S.K.); olga@tsiftsoglou.gr (O.S.T.); 2 Department of Chemistry and Chemical Engineering, Faculty of Agronomy, University of Kragujevac, Cara Dušana 34, 32000 Čačak, Serbia; pavlem@kg.ac.rs

**Keywords:** *Ganoderma adspersum*, sterols, lanostane-type acids, in silico, nuclear receptor, cytotoxicity

## Abstract

*Ganoderma* is a genus of wood-degrading mushrooms with medicinal importance. Most *Ganoderma* species have been studied extensively for their secondary metabolites, biological activities, and ecological value. In this study, the biological activities of the extracts of *G. adspersum* growing wild on *Morus alba* trees in the region of Western Thrace (Greece) were evaluated, and the petroleum ether, dichloromethanolic, and methanolic extracts were studied further for their secondary metabolites. Six substances were isolated by chromatographic (Clumn Chromatography (C.C.), High Performance Liquid Chromatography (HPLC)) and spectroscopic methods (Nuclear Magnetic Resonance (NMR)), which were classified in the following categories: (a) unsaturated fatty acids: *cis-*oleic acid (**1**); (b) sterols: ergosta-7,22-dien-3-one (**2**), ergosta-7,22-dien-3-ol (**3**), and ergosta-5,7,22-trien-3-ol (**4**); and (c) lanostane-type triterpenoids: applanoxidic acid G (**5**) and applanoxidic acid A (**6**). Finally, the biological activities of the extracts were estimated for their antioxidant, antimicrobial, and cytotoxic potential. The methanolic extract of *G. adspersum* showed the highest total antioxidant activity. The results of the antimicrobial activities indicated that all of the extracts had a minimum inhibitory concentration (MIC) ranging between 39.1 and 312.5 μg/mL. The evaluation of the cytotoxic activity of the samples showed once again that the methanolic extract was the most potent among the examined extracts, with half-maximal inhibitory concentration (IC_50_) values of 19.22 μg/mL (Hep2c cells), 32.9 μg/mL (RD cells), and 8.94 μg/mL (L2OB cells). Moreover, the bioactivity scores of the isolated secondary metabolites were calculated using the online computer software program Molinspiration. The compounds showed promising bioactivity scores for drug targets.

## 1. Introduction

*Ganoderma* constitutes a large and diverse genus of wood-decaying basidiomycetes belonging to the Ganodermataceae family [1]. The fungi of this genus grow on the trunks of different deciduous trees, such as oak, willow, elm, acacia, etc. [2]. The genus was first reported and named by Finnish mycologist Petter Adolf Karsten in 1881 [3]. The name arises from the Greek words ”ganos” and ”derma” that signify the shiny skin caused by the morphological characteristics of the fungus [4]. The classification of the genus has been characterized as chaotic due to the difficulty in precisely identifying the miscellaneous features of the species, while some botanists focus on the basidiocarp color for the classification of the mushroom [5,6,7]. 

*Ganoderma* is widespread worldwide, mainly in temperate and tropical climates, specifically in Asia, Africa, and Europe, counting more than 250 species [8,9]. The most popular ones include *G. lucidum*, *G. sinensis*, *G. applanatum*, *G. tsugae*, and *G. atrum* [10]. Nevertheless, only the species *G. lucidum* and *G. sinense* have been accepted from Traditional Chinese Medicine and recognized as “Ling-zhi” by the Chinese or “Reishi” by the Japanese [11]. Despite the therapeutic use of certain *Ganoderma* spp., some species exhibit phytopathogenicity. Only a few species are widely recognized for their phytopathogenicity on both landscape and commercially significant trees. The oil palm tree is harmed by *G. boninense* and *G. zonatum*. [12,13,14].

*Ganoderma* has been thoroughly studied for its medicinal properties, as it has been used traditionally in China since antiquity for the treatment and prevention of various diseases and the promotion of health and longevity [15]. It has also been considered as an “elixir” that represents immortality [16]. The *Pharmacopoeia of China* highlights the beneficial effects of *G. lucidum* in regulating heart function, relieving the symptoms of asthma and dyspnea, boosting the memory, activating the immune system, and handling irregular sleeping. Today, many products derived from *Ganoderma* are consumed in the form of powders, food supplements, and teas [17]. Several reports elucidate the antioxidant, anti-inflammatory, anti-hyperlipidemic, anticancer, anti-aging, antimicrobial, antiviral, hepatoprotective, neuroprotective, and antidepressant properties of various *Ganoderma* species extracts from different parts of the mushroom, both in vitro and in vivo [7,18,19,20,21,22,23,24,25,26,27].

There is a diversity of bioactive constituents and secondary metabolites isolated from *Ganoderma* species, contributing to their therapeutic properties. The major substances identified belong to the classes of polysaccharides, triterpenes, meroterpenoids, sterols, alkaloids, and phenolic compounds. Furthermore, proteins, peptides, vitamins, amino acids, fatty acids, nucleosides, and inorganic elements has also been found [28,29,30]. *Ganoderma lucidum* polysaccharides have exhibited significant pharmacological activity. α- and β-glucans, heteroglucans, peptidoglucans, and polysaccharide–protein conjugates have exerted biological action. They are considered to be potent immunomodulatory agents, as they affect the differentiation and enhance the activity of immune cells (e.g., natural killers, B cells, T cells, dendritic cells, etc.) and act on the release of cytokines (tumor necrosis factor (TNF)-α), interleukins (IL-1β, IL-6), and prostaglandin E, while also displaying antitumor and chemopreventive effects through these mechanisms. 

*Ganoderma adspersum* (Schulz., 1969) Donk (=*G. europaeum* (Stayert, 1961)) is a parasitic species, frequently confused with the species *G. applanatum*, distributed mainly in southern regions of Europe, such as the Balkan Peninsula and Mediterranean Basin, while it is absent from Scandinavian countries [31,32,33]. To date, there are limited reports referring to the phytochemical profile of the species. Tel-Cayan et al. isolated three lanostane-type triterpenes and one steroid from *G. adspersum* extracts [34]. Mayaka and his team isolated three sterol-type triterpenoids from Kenyan mushrooms and studied their antimicrobial activities [35]. The triterpenes and sterol characterized belonged to applanoxidic acid and stigmastane-type derivatives, respectively. In addition, they identified and quantified certain phenolic acids (caffeic acid, rosmarinic acid, ferulic acid, 2,4-dihydrobenzoic acid, and ellagic acid) present in the species. The extracts and the pure compounds were evaluated in vitro for their biological activities. Significant antioxidant and anticholinesterase activities were observed. Specifically, the sterol exhibited remarkable inhibitory effects on lipid peroxidation and butyrylcholinesterase action. Two novel heteropolysaccharides detected in the mushroom, belonging to the class of galactomannans, also indicated potent inhibitory activity [36]. Being a promising therapeutic agent, *G. adspersum* should undergo further study for the protection provided against oxidative stress and Alzheimer’s disease. In addition, the ethanolic extract from a Turkish mushroom was examined for its phenol content and its antioxidant and antimicrobial activities [37].

**Figure 1:**
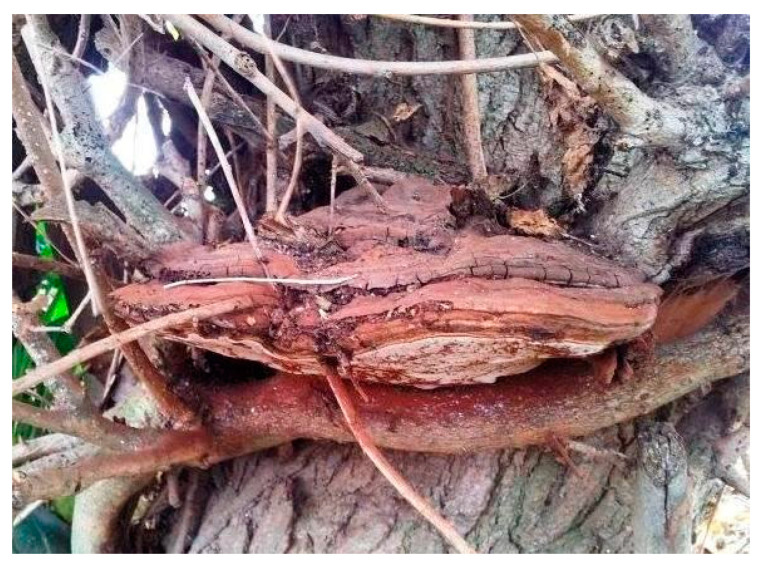
*Ganoderma adspersum* collected from *Morus alba* in order perform the investigation (photo by Msc. Raichan Chafouz).

The aim of this study was the isolation and characterization of mycochemical constituents of wild *Ganoderma adspersum* (Figure 1). Furthermore, the biological activities of extracts produced from the fungus were evaluated as part of this investigation. The fungus was collected from the tree *Morus alba*, grown in the region of Western Thrace, Greece, where it is consumed as a remedy for the treatment of asthma and bronchitis. As the use of *Ganoderma* spp. is rising in Greece, there is a compelling need to investigate the medicinal potential of this mushroom. In this report, we elucidate the extraction and isolation process of six pure compounds derived from *G. adspersum*, and we examine their potential relationships with target receptors (e.g., kinase inhibitors, nuclear receptors, ion-channel modulators) according to the Molinspiration cheminformatics program, assessing their physicochemical properties. Different extracts of the product were also estimated for their total phenolic, flavonoid, and tannin contents and their antioxidant activity in vitro. This study is one of a few to report on the in vitro antimicrobial and cytotoxic effects exerted by *G. adspersum.* Moreover, the results of the current study provide the opportunity for further comparison among the species, as well as the introduction of novel drug candidates derived from this fungus.

## 2. Results

### 2.1. Mycochemical Profile and Total Antioxidant Activity of G. adspersum Extracts

*Ganoderma* is a valuable mushroom genus due to its nutritional and pharmaceutical properties. The mycochemical analysis of species belonging to the genus *Ganoderma* has been demonstrated in recent decades. The mycochemical analysis and antioxidant profile of the extracts from *G. adspersum* are listed in Table 1.

#### Inhibitory Effect against Lipid Peroxidation and ROS Scavenging Activities

As we mentioned above, the methanolic extract of *G. adspersum* was the one with the highest total antioxidant activity. In this study, the inhibitory effect against lipid peroxidation and the scavenging activities against reactive oxygen species (ROS) were measured (Table 2). 

Among the tested extracts, the methanolic extract had the highest phenolic and polyphenolic contents; therefore, it showed the highest antioxidant activity (Table 1). The explanation of these results is related to the polarity of the phenolic compounds (phenolic acids and flavonoids). Higher quantities of phenolic compounds accumulate in polar extracts compered to non-polar extracts. Moreover, it was demonstrated that the methanol extract presented the greatest antioxidant activity and inhibitory effect against lipid peroxidation, followed by the dichloromethanolic extract and, lastly, by the petroleum ether extract. On the other hand, in the case of metal-chelating ability, the non-polar extracts were the most potent (Table 2).

### 2.2. Antimicrobial Activity of G. adspersum Extracts

The inhibitory effects of the *G. adspersum* extracts were evaluated against the bacteria *Staphylococcus aureus*, *Klebsiella pneumoniae*, *Escherichia coli*, *Proteus vulgaris*, *P. mirabilis*, and *Bacillus subtilis* and against the fungi *Candida albicans* and *Aspergillus niger*. As standard antibiotics, we used Amracin as an antibacterial and nystatin as an antifungal (Table 3).

Concerning the antimicrobial activity (Table 3), the results indicate that all of the examined extracts expressed weak antimicrobial activity, with a range of minimum inhibitory concentrations (MICs) between 39.1 and 312.5 μg/mL. The best recorded MICs of the petroleum ether extract were those against *Escherichia coli*, with an MIC of 39.1 μg/mL, and *Aspergillus niger*, with an MIC of 78.1 μg/mL. On the other hand, the dichloromethanolic extract showed remarkable effects against *Staphylococcus aureus* and *Candida albicans*, with an MIC of 39.1 μg/mL, and also against *Escherichia coli* and *Proteus vulgaris*, with an MIC of 78.1 μg/mL. Finally, the methanolic extract was most potent against *Staphylococcus aureus*, with an MIC of 39.1 μg/mL, and against *Klebsiella pneumoniae*, *Escherichia coli*, *Proteus mirabilis*, and *Bacillus subtilis* with an MIC of 78.1 μg/mL. These results are consistent with previous reports [35,36].

### 2.3. Cytotoxic Activities of G. adspersum Extracts

The cytotoxic effects of the *G. adspersum* extracts were evaluated in vitro against the cell lines Hep2c (human cervix carcinoma), RD (human rhabdomyosarcoma), and L2OB (murine fibroblasts) (Table 4).

### 2.4. Characterization of Isolated Compounds

On the basis of ^1^H, ^13^C, and 2D NMR (gDQCOSY, gHSQCAD, and gHMBCAD, respectively) spectroscopic analysis, and through comparison with the literature data, the chemical structures of six isolated compounds were established (Figure 2): *cis*-oleic acid (**1**) [38], ergosta-7,22-dien-3-one (**2**) [39], ergosta-7,22-dien-3-ol (**3**) [40], ergosta-5,7,22-trien-3-ol (**4**) [35], applanoxidic acid G (**5**) [41], and applanoxidic acid A (**6**) [42]. The structures of the isolates are given in Figure 2 and were elucidated based on 1D and 2D NMR spectral analyses (see the Supplementary Materials: Tables S1–S5 and Figures S1–S25). 

### 2.5. In Silico Analysis of the Secondary Metabolites Isolated from G. adspersum

Utilizing computational chemistry methods to examine sizable chemical databases in order to find potential novel drug candidates is known as virtual screening or in silico screening. From basic virtual screening methods that check for the presence or absence of specified substructures or matches in calculated chemical characteristics, to complex virtual docking procedures designed to fit potential ligand molecules into the target receptor site, there are many different virtual screening techniques available [43]. The results of the secondary metabolites isolated from *G. adspersum* are listed in Table 5. 

## 3. Discussion

Many studies have been conducted to promote *Ganoderma* extracts as an adjuvant remedy for various diseases. The pharmacological potential of this mushroom has led to its nomination as the greatest adaptogen in nature and has triggered the interest for constant investigation, aiming at the isolation and characterization of its secondary metabolites [44]. Moreover, they have been tested for their antioxidant and antimetastatic properties [45,46]. Triterpenoids constitute the main bioactive compounds of this fungus [47].

To the best of our knowledge, there are not many studies concerning the polyphenol composition of *G. adspersum* and its biological activities. According to Shomali et al., ethanolic extracts show high contents of both flavonoids and phenolics, which are related to their antioxidant activity [37]. In another study, Raks et al. performed various extraction methods in six different *Ganoderma* spp. collected from trees in Turkey and investigated their antioxidant activities [48]. The same study reported the results of the total phenolic and flavonoid contents. Apart from the extraction method, another parameter that effects the amounts of total phenolics and flavonoids (and, thus, the bioactivity of the *Ganoderma* extracts) seems to be the origin of the fungus (i.e., wild or cultivated). The difference in that case was that the mushroom material was not wild but from mycelial cultures. The results of recent studies confirm the aforementioned conclusion [49,50]. Furthermore, it has also been reported that the freshness (i.e., fresh or dry) of the mushroom material plays a key role in the amounts of bioactive components recovered [51]. As was expected, the mycochemical analysis of our wild mushroom material showed that the presence of total phenolics, flavonoids, condensed tannins, and gallotannins increased as the solvent polarity increased. 

Researchers’ interest in *Ganoderma* extracts’ antitumor properties has grown over time. The antitumor action is associated with increasing the host cell’s immunological regulatory function and encouraging tumor cell death [4]. In the present study, we examined the cytotoxic effects of *G. adspersum* extracts. The results proved once more that the methanolic extract was the most potent (among the tested extracts), with half-maximal inhibitory concentration (IC_50_) values of 19.22 μg/mL, 32.9 μg/mL, and 8.94 μg/mL against Hep2c, RD, and L2OB cells, respectively, followed by the dichloromethanolic extract and, finally, by the petroleum ether extract (Table 4). In terms of the basic criterion for the cytotoxic activity of a plant extract according to the American National Cancer Institute (i.e., activity < 30 µg/mL), the methanolic extract proved to be active against the cancer cell lines Hep2c (19.22 ± 0.93 μg/mL) and L2OB (8.94 μg/mL) [52]. 

More than 300 triterpenes have been isolated from *Ganoderma* species, belonging to the class of lanostanes. Lanostane-type triterpenes have been examined for their cytotoxicity against different cancer cell lines, including lung, colon, pancreas, and breast cancers. These compounds, including the characteristic ganoderic acids, downregulated the proliferation and growth cycle of the tumor cells [53]. Even in silico studies have demonstrated their antiproliferative effects through the interaction with nuclear receptors such as the vitamin D receptor, diminishing the cell growth and activating cell differentiation [54]. The androgenic outcomes of lanostanoids have been indicated by another in silico analysis explaining the affinity between the C3- ketonic group of the triterpenes and the androgen receptor belonging to the nuclear receptor superfamily [55]. All of the abovementioned reports explain the high bioactivity scores that all of the isolated secondary metabolites expressed in the in silico study of the present research. It seems that the structures of the lanostanoids and sterols play a key role in the case of nuclear receptor ligands. Secondary metabolites isolated from *G. adspersum* showed promising bioactivity scores for drug targets according to the Molinspiration software, e.g., for nuclear receptor ligands (compounds **2**, **3**, **4**, **5**, and **6**). It must be noted that all of the reported compounds seem to be less effective as kinase inhibitors (Table 5).

All of the secondary metabolites isolated from the extracts of *G. adspersum* were triterpenoids. The mycochemical analysis led to the isolation of three sterols (compounds **2**–**4**) and two lanostanoids (compounds **5** and **6**). This also underlines the lanostanoids’ antimycobacterial and anti-complement activity [56,57]. Reports also suggest their inhibitory action against angiotensin-converting enzyme, α-glucosidase, and cholinesterase [58,59]. In addition, ergostane-type sterols have been isolated and identified from *Ganoderma* species. These metabolites are known for their anti-inflammatory activity in suppressing lipopolysaccharide-induced inflammation and reducing the expression of cyclooxygenase-2 and tumor necrosis factor (TNF-α). Therefore, they may be investigated for the treatment of chronic ailments associated with the above pathophysiological conditions [60].

*Cis*-oleic acid (**1**) is a well-known unsaturated free fatty acid that is widely distributed in plants and animals [61]. It is a primary metabolite, and its presence was expected. There are various bibliographic data reporting the presence of fatty acids in *Ganoderma* spp. [62,63].

Compounds **2**, **3**, and **4** are triterpenoids with a steroidal structure. Ergosterols form the basic membrane lipids of fungi [36,37]. Ergosta-5,7,22-trien-3-ol (**4**) is commonly known as ergosterol and has been isolated from many *Ganoderma* spp., such as *G. lucidum* [64], *G. colossum* [65], *G. lipsiense, G. applanatum*, *G. australe*, and *G. fornicatum* [66]. According to a recent study, ergosterol showed possible inhibitory effects against MDA-MB-231 (epithelial human breast cancer cells), HepG2 (human hepatoma cells), and HUVECs (human umbilical vein endothelial cells), indicating that it has antitumor and anti-angiogenesis properties. It must be mentioned that there was no evident cytotoxicity against normal cells according to the literature [64].

Ergosta-7,22-dien-3β-ol (**3**) is also isolated from many *Ganoderma* spp., such as *G. lucidum*, *G. amboinense*, *G. carnosum*, *G. tsugae*, *G. applanatum*, *G. neo-japonicum*, *G. lipsiense, G. australe*, *G. annulare*, *G. pfeifferi*, and *G. lucidum [*66]. Ergosta-7,22-dien-3-one (**2**) is also a very abundant compound in the genus *Ganoderma* and has been reported in *G. australe*, *G. lucidum*, *G. applanatum*, *G. neo-japonicum*, *G. lipsiense, G. concinna* [16], *G. oerstedii* [66], and *G. concinnum* [67]. 

Compounds **5** and **6** are lanostane-type triterpenes, one of the main classes of bioactive components of *Ganoderma* spp. [68]. The name of compound **6** (applanoxidic acid A) was given after *Ganoderma applanatum*, which was the fungus from which applanoxidic acids were isolated for the very first time in 1991 by Tokuyama and his colleagues [42]. Some years later, the members of the same team isolated applanoxidic acid G (**5**), among other compounds [41]. To date, these two secondary metabolites have also been isolated from *G. annulare*, *G. pfeifferi*, and *G. australe* [16]. According to the literature, both applanoxidic acids A and G exert inhibitory effects on EBV-EA activation and cytotoxicity against HL-60 cells. Also, applanoxidic acid A has antifungal activity against the growth of *Micronosporum cannis* and *Trichophyton mentagrophyte* [47]. 

## 4. Materials and Methods

### 4.1. General Experimental Procedures

Column chromatography (CC) was carried out on silica gel 60 (Merck Art. 9385, Darmstadt, Germany) with gradient elution, with the solvent mixtures indicated in each case. 

Vacuum liquid chromatography (VLC) was carried out on silica gel 60 H (Merck Art. 7736) with gradient elution, with the solvent mixtures indicated in each case. Thin-layer chromatography (TLC) was carried out on silica gel plates (Kieselgel F254, Merck, Art. 5554), and detection was carried out on TLC plates under UV light (absorbance: 254 and 366 nm). For the visualization of the chromatograms on silica gel, vanillin–H_2_SO_4_ spray reagent was used. For high-performance liquid chromatography (HPLC), a Lab Alliance Series III pump (LabAlliance, Scientific Systems, Inc., 349 N Science Park Rd., State College PA 16803) equipped with Clarity software (version 9.0.) and a Shodex RI-101 Detector (Kawasaki, Japan) was used, using a C18 ODS1 Spherisorb with a 10 µm column that measured 250 mm × 10 mm (Waters). Spectroscopic NMR data: The ^1^H-NMR and ^13^C-NMR spectra were recorded in CD_3_OD and CDCl_3_ using an AGILENT DD2 500 (500.1 MHz for ^1^H-NMR and 125.5 MHz for ^13^C-NMR) spectrometer. The chemical shifts are provided in δ (ppm) values relative to TMS (CD_3_OD: 3.31 ppm for ^1^H-NMR and 49.05 ppm for ^13^C-NMR; CDCl_3_: 7.26 ppm for ^1^H-NMR and 77.6 ppm ^13^C-NMR).

Mushroom material: *G. adspersum* (Schulz.) Donk. was collected from Heliopetra in the municipality of Topiros, Xanthi, Greece and was identified by Dr. Zacharoula Gkonou at the Department of Biology of the National and Kapodistrian University of Athens (Athens, Greece).

### 4.2. Extraction and Isolation

#### 4.2.1. Extraction

The naturally air-dried (in a shady place) fruiting parts of *G. adspersum* (562.0 g) were finely ground and exhaustively extracted at room temperature with petroleum ether (40 °C–60 °C) (PE), dichloromethane (DM), methanol (M), and a mixture of methanol:water (MW) (70:30), respectively. In each case, the extract was filtered and evaporated in vacuum. In this study, the biological activities and total phenolic compounds of the first three extracts were evaluated.

#### 4.2.2. Compound Isolation

The petroleum ether extract (GPE) (1.46 g) was subjected to column chromatography (CC) on silica gel (17 × 3.5 cm), using increasingly polar eluent systems of hexane (He), dichloromethane (DM), and methanol (M). Eventually, 39 fractions (GPE-A to GPE-ZO) were obtained in total. Fraction GPE-V (196.6 mg, eluted with DM:M 99.5:0.5) consisted of a mixture of compounds **3** (ergosta-7,22-dien-3-ol) and **4** (ergasta-5,7,22-trien-3-ol). Fraction GPE-ZA (32.1 mg, eluted with DM:M 98.5:1.5) was identified as compound **1** (*cis*-oleic acid). Fraction GPE-Q (49.9 mg), eluted with the less polar solvent systems He:DM 20:80 and DM 100%, was characterized as compound **2** (ergosta-7,22-dien-3-one). In addition, the dichloromethane (GDM) extract (1.19 g) was subjected to CC on silica gel (15 × 3.5 cm). Augmenting the polarity of the solvent systems composed of He, DM, and M, this chromatographic separation led to 24 fractions (GDM-A to GDM-Y). Fraction GDM-R (42.6 mg, eluted with DM:M 92:8) was further fractionated by semi-preparative HPLC, applying isocratic elution (acetonitrile:water, 3:2, 1.00 mL/min) and resulting in the isolation of compound **6** (applanoxidic acid A, tR = 33.8 min, 8.3 mg). Fraction GDM-S (32.4 mg, eluted with DM:M 90:10) was purified by semi-preparative HPLC (isocratic elution, acetonitrile:H_2_O, 2:3, 1.00 mL/min), allowing for the isolation of compound **6** (applanoxidic acid A, tR = 33.5 min, 5.9 mg). Fraction GDM-C (373.6 mg) was subjected to CC on silica gel (45 × 2.5 cm). Mixtures of DM and M of increasing polarity were considered suitable for the elution of 31 fractions (GDM-CA to GDM-CZF). GDM-CP (21.3 mg) was eluted with both DM:M 98:2 and DM:M 95:5 and then identified as compound **3** (ergostan-7,22-dien-3-ol). The methanolic GM extract (11.74 g) was separated by VLC (vacuum liquid chromatography) on silica gel (10 × 7 cm), utilizing mixtures of DM, M, and H_2_O as eluents. During the separation, the polarity of the mobile phase was increased, yielding 13 fractions (GM-A to GM-N) of approximately 300 mL each. The GM-A fraction (2.38 g), eluted with DM 100%, was subjected to CC on silica gel (18 × 3.5 cm). The elution strength was increased gradually, using mixtures of the following solvents: DM, M, and H_2_O. A total of 25 fractions of 30 mL each were collected (GM-AA to GM-AZ). The GM-AK fraction (47.2 mg, eluted with DM:M:H_2_O 90:10:1) was further purified by semi-preparative HPLC (isocratic elution, acetonitrile:H_2_O, 2:3, 1.00 mL/min), resulting in the identification of compound **5** (applanoxidic acid G, tR = 46.3 min, 4.8 mg). 

### 4.3. Antimicrobial Activity

The antimicrobial activity of the extracts was tested in vitro against the bacteria *Staphylococcus aureus* ATCC 25923, *Klebsiella pneumoniae* ATCC 13883, *Escherichia coli* ATCC 25922, *Proteus vulgaris* ATCC 13315, *Proteus mirabilis* ATCC 14153, and *Bacillus subtilis* ATCC 6633, as well as the fungi *Candida albicans* ATCC 10231 and *Aspergillus niger* ATCC 16404. The experiments were performed according to the method of Mirković et al. (2022) [69]. 

#### Minimum Inhibitory Concentrations (MICs)

The minimum inhibitory concentration (MICs) of the extracts and cirsimarin against the test bacteria were determined by microdilution in 96-well microliter plates according to the method of Satyajit et al. (2007) [70]. The MIC value was determined to be the lowest concentration at which a color change occurred. The MICs for the investigated chemicals and the reference medicines (Amracin for bacteria and nystatin for fungi) were determined by averaging three results. The results were expressed in μg/mL [71].

### 4.4. Measurement of Cytotoxic Activity by MTT Assay

The influence of the sample extracts on the growth of malignantly transformed cell lines was evaluated by the MTT assay. The following cell lines were used (cell lines were donated from the collection of cell lines of the Institute of Virology, Vaccines, and Serums “Torlak”, Belgrade, Serbia): RD (substrate: MEM Eagle/10% FCS) (cell line derived from human rhabdomyosarcoma), Hep2c (medium: MEM Eagle/5% FCS) (cell line derived from human cervix carcinoma—HeLa derivative), and L2OB (medium: MEM Eagle/10% FCS) (cell line derived from murine fibroblasts). The identification of the examined tumor cells was confirmed by the Diagnostics Laboratory of the Torlak Institute in Belgrade, Serbia.

The measurements are represented as the percentage of positive control growth, with *cis*-diamminedichloroplatinum (*cis*-DDP) determined in positive control wells being taken as 100% growth [72,73,74]. According to the American National Cancer Institute (NCI), the criterion of cytotoxic activity for plant extracts is IC_50_ < 30 µg/mL [52]. All experiments were conducted in triplicate.

### 4.5. Determination of the Secondary Metabolite Contents

#### 4.5.1. Determination of Total Phenolic Contents

Total phenols were estimated according to the Folin–Ciocâlteu method of Singleton et al. [75]. 

#### 4.5.2. Determination of Total Flavonoid Contents

Total flavonoids were determined as described by Brighente et al. [76]. 

#### 4.5.3. Determination of Condensed Tannins

The method for the determination of condensed tannins relied on the precipitation of proanthocyanidins with formaldehyde according to the method of Verrmeris and Nicholson (2006) [77]. 

#### 4.5.4. Determination of Condensed Gallotannins

Gallotannins are hydrosoluble tannins containing a gallic acid residue esterified to a polyol. Gallotannins can be detected quantitatively by the potassium iodate assay. The interaction of potassium iodate (KIO_3_) with galloyl esters produces a red intermediate and, eventually, a yellow product. The method used was the one described by Verrmeris and Nicholson in 2006 [77]. 

### 4.6. Antioxidant Activity

#### 4.6.1. Determination of Total Antioxidant Capacity

The total antioxidant activity of the *Ganoderma adspersum* extracts was evaluated by the phosphomolybdenum method as described by Prieto et al. (1999) [78]. 

#### 4.6.2. Determination of DPPH Free Radical Scavenging Activity

The method used by Takao et al. [79] was adopted, with suitable modifications from the work of Kumarasamy et al. [80]. 

#### 4.6.3. Determination of Hydroxyl Radical Scavenging Activity

The ability of the extracts derived from *Ganoderma adspersum* extracts to inhibit non-site-specific hydroxyl-radical-mediated peroxidation was determined according to the method described by Hinneburg et al. [81]. 

#### 4.6.4. ABTS Radical Scavenging Assay

The antioxidant capacity was estimated in terms of the ABTS^●+^ radical scavenging activity, following the procedure described by Delgado-Andrade et al. [82]. 

#### 4.6.5. Determination of the Inhibitory Activity toward Lipid Peroxidation

The antioxidant activity of the extracts from *G. adspersum* was determined by the thiocyanate method, as described by Hsu et al. [83].

#### 4.6.6. Measurement of Ferrous-Ion-Chelating Ability

The ferrous-ion-chelating activity of the three extracts was measured by the decrease in absorbance of the iron(II)–ferrozine complex at 562 nm, as described by Carter and Yan et al. [84,85]. 

### 4.7. In Silico Study

SMILES notations of all of the isolated secondary metabolites were fed into the online Molinspiration software, version 2011.06 (accessed on 14 January 2023) (www.molinspiration.com), for the prediction of bioactivity scores for drug targets (GPCR ligands, kinase inhibitors, ion-channel modulators, enzymes, and nuclear receptors). 

## 5. Conclusions

The mycochemical investigation of *Ganoderma adspersum* proved that this species is a rich source of triterpenoids such as sterols and lanostanoids. We extracted and characterized six known compounds (**1**–**6**) from the fruiting parts of wild *G. adspersum*. Moreover, this study investigated the phenolic contents of extracts from *G. adspersum*, proving that the highest concentrations of phenolics, flavonoids, condensed tannins, and gallotannins appeared in the methanol extract in comparison to the non-polar extracts (i.e., dichloromethane and petroleum ether), which expressed higher metal-chelating activity. Also, the methanolic extract exhibited the strongest antioxidant activity and inhibitory effect against lipid peroxidation. None of the examined extracts expressed satisfactory antimicrobial activity. It is not surprising that the polar extract of *Ganoderma* showed the best results for cytotoxicity against the examined cancer cell lines. It is known that the compounds biosynthesized from various *Ganoderma* species are valuable candidates for anticancer activity. The in silico analyses of the isolated secondary metabolites of *G. adspersum* were in accordance with the bibliographical data. 

## Figures and Tables

**Figure 2:**
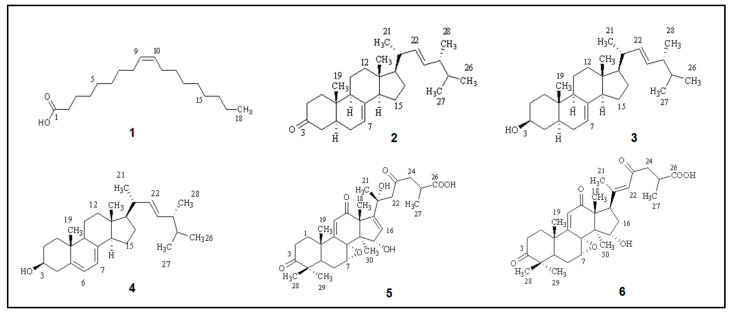
Isolated compounds from *Ganoderma adspersum*.

**Table 1:** Mycochemical profile and antioxidant activity of *G. adspersum* extracts.

Sample	Total Phenolics (mg GA/g)	Flavonoids (mg RU/g)	Condensed Tannins (mg GA/g)	Gallotannins (mg GA/g)	Total Antioxidant Capacity (μg AA/g)
GM	67.87 ± 0.27	43.41 ± 1.13	45.42 ± 0.67	31.12 ± 0.33	87.14 ± 0.99
GDM	59.25 ± 0.55	37.86 ± 0.37	33.65 ± 0.94	26.45 ± 0.14	71.05 ± 1.25
GPE	38.06 ± 0.06	20.79 ± 0.70	25.23 ± 0.53	19.06 ± 0.43	50.54 ± 1.03

GPE: *G. adspersum* petroleum ether extract; GDM: *G. adspersum* dichloromethane extract; GM: *G. adspersum* methanol extract. Results are the mean values ± SD from three experiments.

**Table 2:** Inhibitory effect against lipid peroxidation and ROS scavenging activities.

Sample	DPPH Scavenging Activity	Inhibitory Effect against Lipid Peroxidation	Metal-Chelating Activity	Hydroxyl Radical Scavenging Activity	ABTS Radical Scavenging Assay
	IC50 (μg/mL)				
GM	21.45 ± 0.87	28.64 ± 0.31	45.26 ± 0.14	37.59 ± 0.95	25.71 ± 0.69
GDM	43.07 ± 0.99	41.34 ± 0.84	40.20 ± 0.36	69.18 ± 0.79	38.20 ± 1.04
GPE	46.79 ± 0.73	47.09 ± 0.58	40.14 ± 0.57	85.99 ± 0.86	51.10 ± 1.02
Gallic acid	3.79 ± 0.69	255.43 ± 11.68	-	59.14 ± 1.10	1.96 ± 0.41
Ascorbic acid	6.05 ± 0.34	>1000	-	160.55 ± 2.31	10.98 ± 0.95
BHT	15.61 ± 1.26	1.00 ± 0.23	-	33.92 ± 0.79	7.23 ± 0.87
α-Tocopherol	-	0.48 ± 0.05	-	-	-

GPE: *G. adspersum* petroleum ether extract; GDM: *G. adspersum* dichloromethane extract; GM: *G. adspersum* methanol extract. Results are the mean values ± SD from three experiments.

**Table 3:** Antimicrobial activities of *G. adspersum* extracts.

Microbial Strains	GM	GDM	GPE	A	N
	MIC (μg/mL)				
*Staphylococcus aureus* ATCC 25923	39.1 ± 0.06	39.1 ± 0.06	312.5 ± 0.49	0.97 ± 0.24	-
*Klebsiella pneumoniae* ATCC 13883	78.125 ± 0.49	156.25 ± 0.97	312.5 ± 0.03	0.49 ± 0.06	-
*Escherichia coli* ATCC 25922	78.125 ± 0.24	78.125 ± 0.24	39.1 ± 0.06	0.97 ± 0.03	-
*Proteus vulgaris* ATCC 13315	312.5 ± 0.12	78.125 ± 0.12	312.5 ± 0.97	0.49 ± 0.015	-
*Proteus mirabilis* ATCC 14153	78.125 ± 0.97	156.25 ± 0.24	312.5 ± 0.49	0.49 ± 0.24	-
*Bacillus subtilis* ATCC 6633	78.125 ± 0.49	312.5 ± 0.49	312.5 ± 0.24	0.24 ± 0.06	-
*Candida albicans ATCC 10231*	156.25 ± 0.06	39.1 ± 0.06	312.5 ± 1.95	-	1.95 ± 0.24
*Aspergillus niger* ATCC 16404	312.5 ± 0.12	156.25 ± 0.03	78.125 ± 0.97	-	0.97 ± 0.12

GPE: *G. adspersum* petroleum ether extract; GDM: *G. adspersum* dichloromethane extract; GM: *G. adspersum* methanol extract; A: Amracin, N: nystatin. Results are the mean values ± SD from three experiments.

**Table 4:** Cytotoxic activities of *G. adspersum* extracts.

Sample	Hep2c Cells	RD Cells	L2OB Cells
	IC50 (μg/mL)		
GM	19.22 ± 0.93	32.99 ± 4.73	8.94 ± 0.85
GDM	28.69 ± 0.51	55.51 ± 1.99	21.25 ± 1.06
GPE	42.83 ± 0.46	79.65 ± 0.39	36.25 ± 0.57
*cis*-platin	0.94 ± 0.55	1.4 ± 0.97	0.72 ± 0.64

GPE: *G. adspersum* petroleum ether extract; GDM: *G. adspersum* dichloromethane extract; GM: *G. adspersum* methanol extract. Results are the mean values ± SD from three experiments.

**Table 5:** Bioactivity scores for drug targets according to Molinspiration software.

Compound	GPCR Receptor	Ion-Channel Modulator	Kinase Inhibitor	Nuclear Receptor Ligand	Protease Inhibitor
(**2**) Ergosta-7,22-dien-3-one	0.05	−0.03	−0.56	0.59	−0.08
(**3**) Ergosta-7,22-dien-3-ol	0.20	0.12	−0.27	0.66	0.05
(**4**) Ergosta-5,7,22-trien-3-ol	0.14	−0.14	−0.40	0.82	−0.15
(**5**) Applanoxidic acid G	0.19	−0.20	−0.53	0.67	0.17
(**6**) Applanoxidic acid A	0.11	−0.21	−0.67	0.80	0.05

The best score is around 1.

## Data Availability

Data are contained within the article and Supplementary Materials.

## Supplementary Materials

The supporting information can be downloaded at: https://www.mdpi.com/article/10.3390/ijms25010516/s1.
