# Supplementary material for: Ganoderma adspersum (Ganodermataceae): Investigation of Its Secondary Metabolites and the Antioxidant, Antimicrobial, and Cytotoxic Potential of Its Extracts
Source: Int J Mol Sci. 2023 Dec 30;25(1):516. doi: 10.3390/ijms25010516 (PMC10779304; doi:10.3390/ijms25010516)
Supplement: Supplementary file 1 [file ijms-25-00516-s001.zip › ijms-2689172-supplementary.pdf]

Supplement material

Ganoderma adspersum (Ganodermataceae): Investigation of its secondary metabolites and the anti-oxidant, anti-microbial and cytotoxic potent its extracts

Raichan Chafouz [1], Sofia Karavergou [1], Olga St. Tsiftsoglou [1], Pavle Z. Maskovic [2] and Diamanto Lazari [1,*]

[1]Laboratory of Pharmacognosy, School of Pharmacy, Faculty of Health Sciences, Aristotle University of Thessaloniki, 54124 Thessaloniki, Greece

[2]Department of Chemistry and Chemical Engineering Department of Food Technology, Faculty of Agronomy, University of Kragujevac, Cara Dušana 34, 32000 Čačak, Republic of Serbia. Phone: +381 32 30 34 00; pavlem@kg.ac.rs (P.Z.M.)

*Correspondence: D.L, dlazari@pharm.auth.gr; Tel.: 0030 2310997617

Abstract: *Ganoderma* is a genus of wood degrading mushrooms with medicinal importance. Most of *Ganoderma* species have been studied extensively for their secondary metabolites, biological activities and ecological value. In this study, biological activities of the extracts of *G. adspersum*, growing wild on *Morus alba* tree in the region of Western Thrace (Greece) have been evaluated, as well as the petroleum ether, dichloromethanolic and methanolic extracts were studied further for their secondary metabolites. So far, six substances have been isolated by chromatographic (C.C, HPLC) and spectroscopic methods (NMR), which were classified in the following categories a) unsaturated fatty acid: cis-oleic acid (**1**), b) sterols: ergosta-7,22-dien-3-one (**2**), ergosta-7,22-dien-3-ol (**3**), ergosta-5,7,22-trien-3-ol (**4**) and c) lanostane-type triterpenoids: applanoxidic acid G (**5**) and applanoxidic acid A (**6**). Finally, the biological activities of the extracts were estimated for their antioxidant, anti-microbial and cytotoxic potent. The methanolic extract of *G. adspersum* shows the highest total anti-oxidant activity. The results of the antimicrobial activities indicate that all the extracts had a range of minimum inhibitory concentration (MIC) between 39.1-312.5 μg/mL. The evaluation of cytotoxic activity of the samples showed once again that the methanolic extract was the most potent among the examined extracts with half-maximal inhibitory concentration (IC50) 19.22 μg/mL (Hep2c cells), 32.9 μg/mL (RD cells), 8.94 μg/mL (L2OB cells), respectively. Moreover, bioactivity score of the isolated secondary metabolites were calculated by online computer software program Molinspiration. Compounds showed promising bioactivity scores for drug targets.

Keywords: *Ganoderma adspersum*; sterols; lanostane type acids; *in silico*; nuclear receptor; cytotoxicity

Table S1. NMR spectroscopic data of compound **1**, ($CDCl_3$, 500 MHz)

No	δ_C	Type C	δ_H	H	Multiplicity (Hz)
1	189.1	COOH	-	-	o.s
2	34.0	CH_2	2.31	2	o.s
3	24.7	CH_2	1.61	2	o.s
4	27.9	CH_2	1.25	2	o.s
5	27.9-27.1	CH_2	o.s	2	o.s
6	27.9-27.1	CH_2	o.s	2	o.s
7	27.9-27.1	CH_2	1.25	2	o.s
8	27.2	CH_2	2.05	2	o.s
9	130.0	CH	5.34	1	o.s
10	129.7	CH	5.34	1	o.s
11	27.1	CH_2	2.05	2	o.s
12	27.9-27.1	CH_2	1.25	2	o.s
13	27.9-27.1	CH_2	o.s	2	o.s
14	27.9-27.1	CH_2	o.s	1	o.s
15	27.9-27.1	CH_2	o.s	1	o.s
16	32.0	CH_2	o.s	1	o.s
17	22.7	CH_2	1.25	2	o.s
18	14.1	CH_3	0.85	3	t

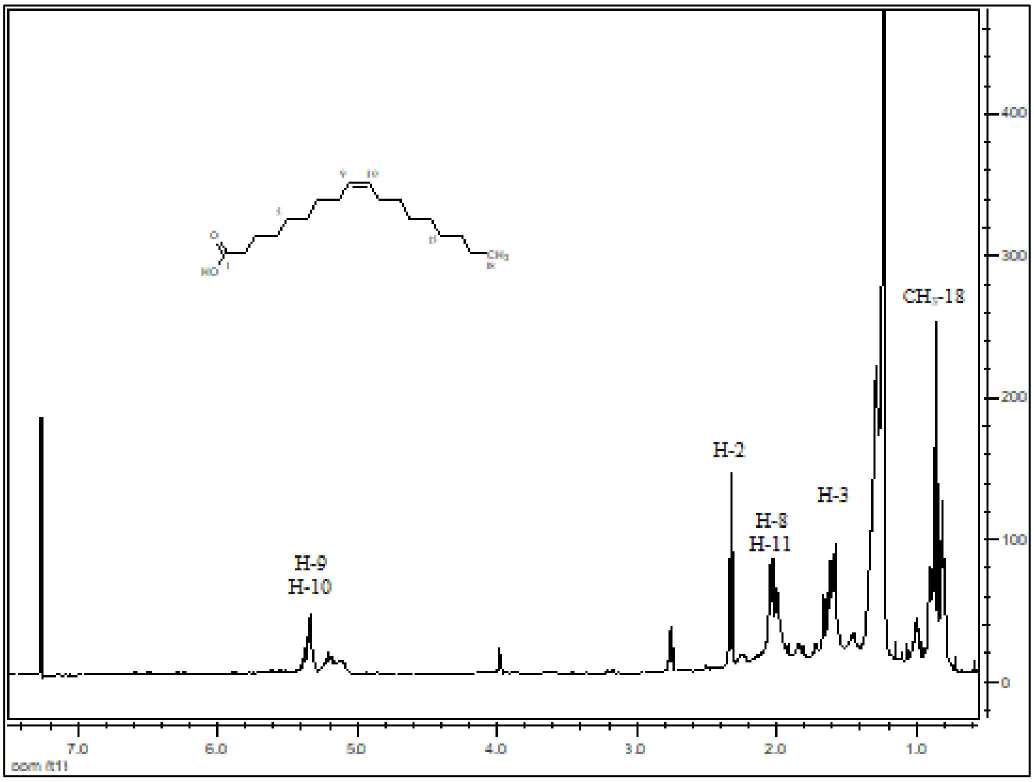

Figure S1. ^{1}H-NMR spectrum of compound **1** ($CDCl_3$, 500MHz)

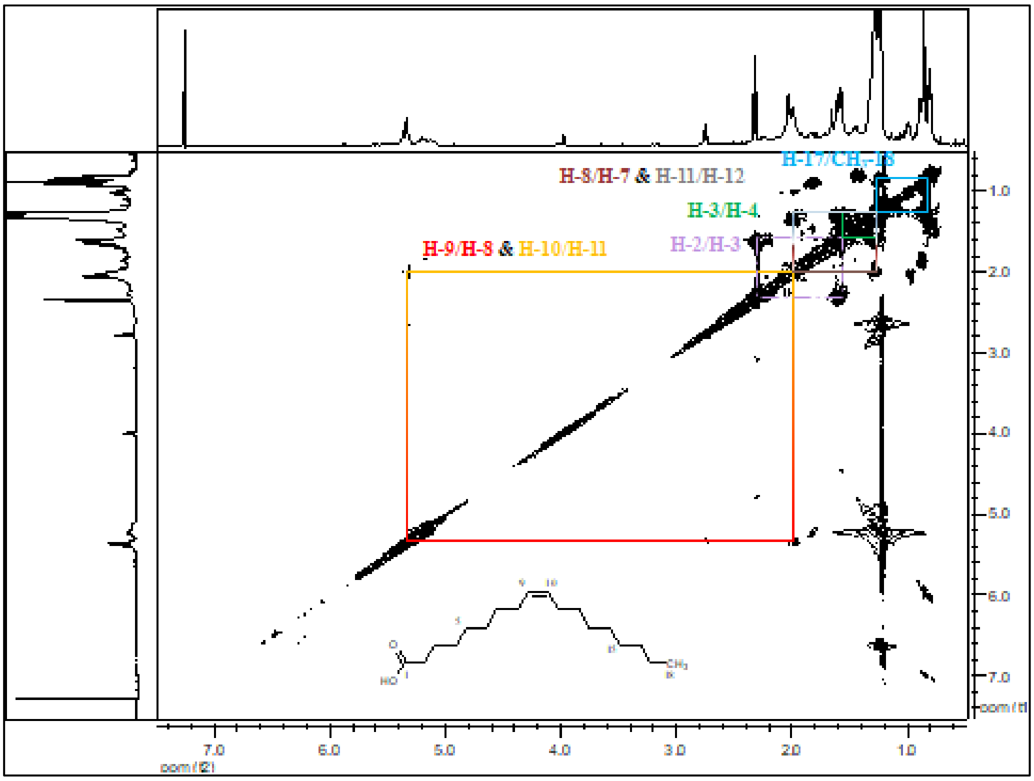

Figure S2. gDQCOSY spectrum of compound **1** ($CDCl_3$, 500MHz)

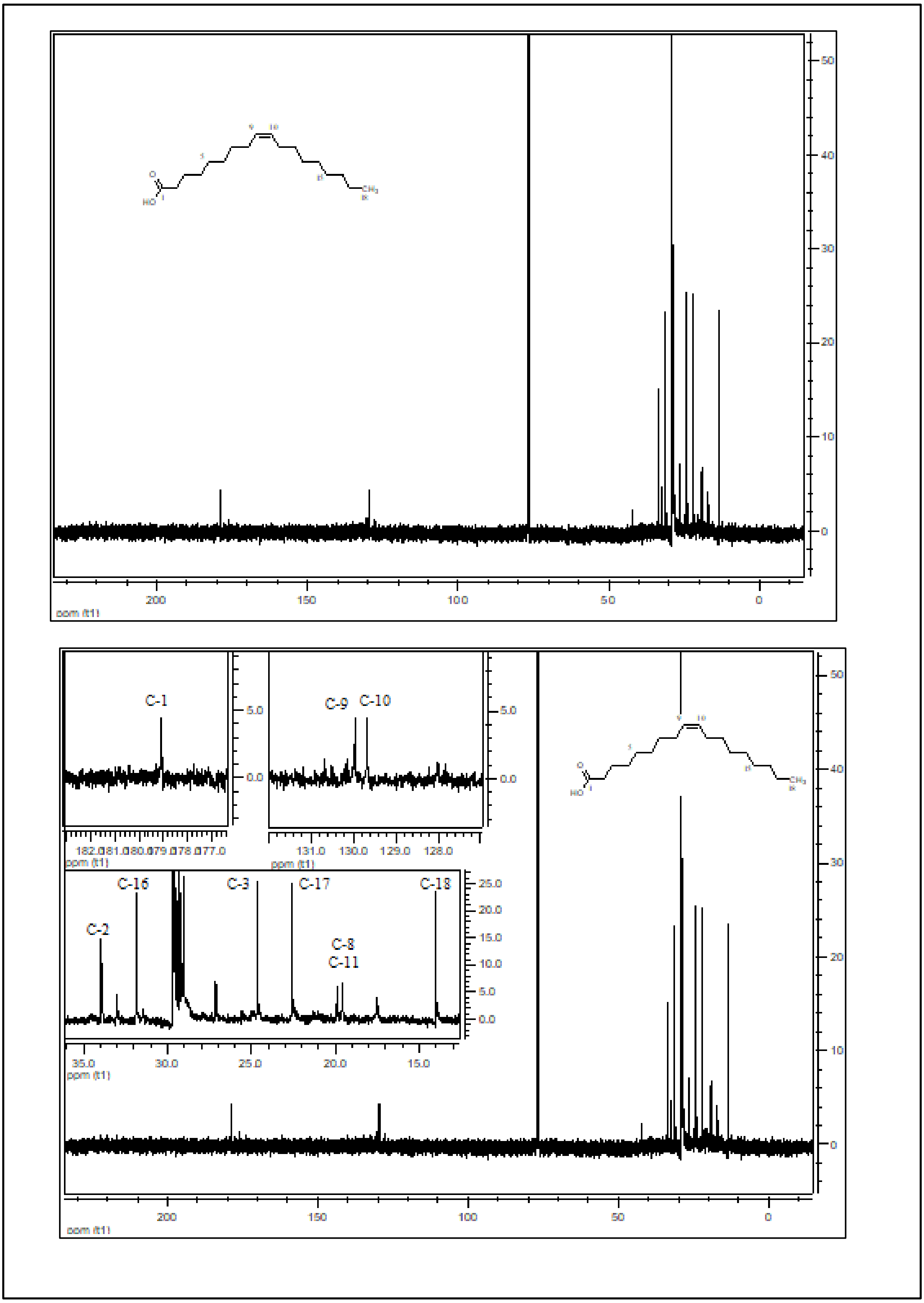

Figure S3. ^{13}C-NMR spectrum of compound **1** ($CDCl_3$, 125MHz)

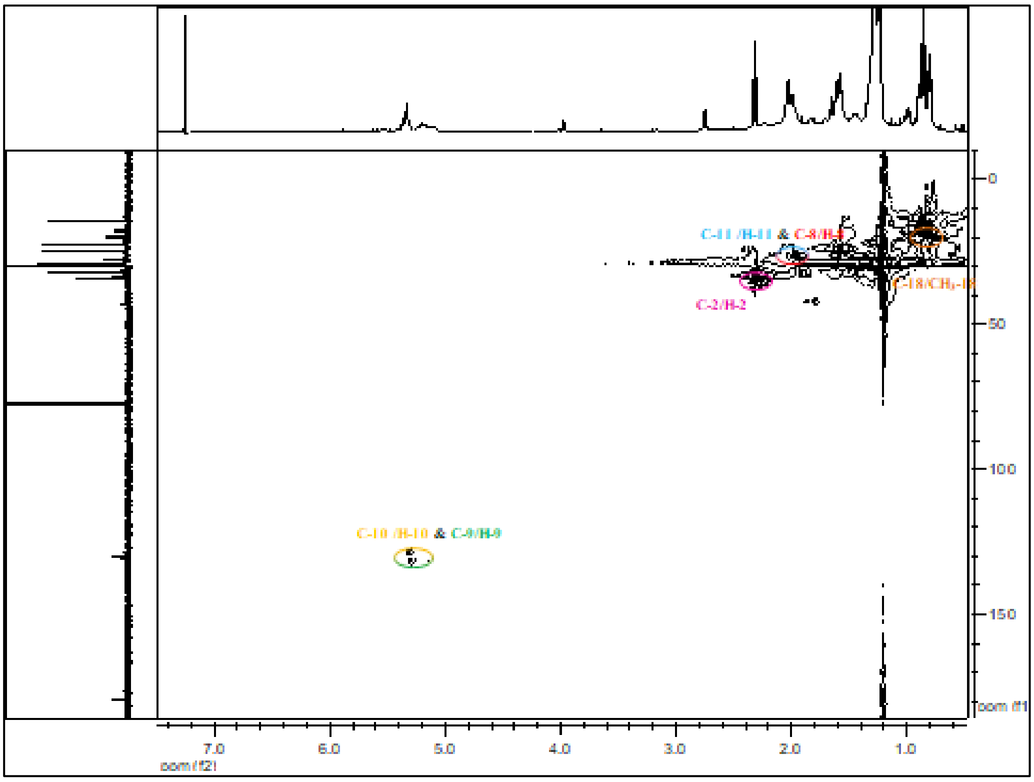

Figure S4. gHSQCAD spectrum of compound **1** ($CDCl_3$, 500MHz)

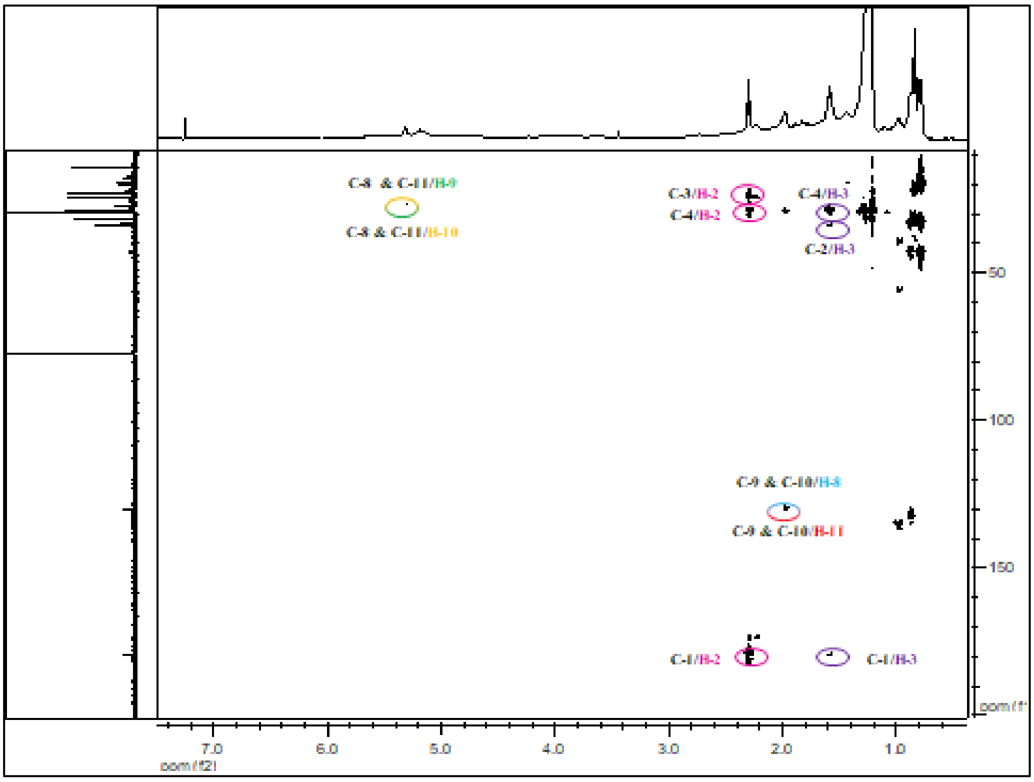

Figure S5. gHMBCAD spectrum of compound **1** ($CDCl_3$, 500MHz)

Table S2. NMR spectroscopic data of compound **2**, (CD_3OD, 500 MHz/125MHz)

No	δ_C	Type C	δ_H	H	Multiplicity J (Hz)
1a	38.8	CH_2	2.10	1	o.s
1b			1.98	1	o.s
2a	38.1	CH_2	2.38	1	o.s
2b			2.25	1	o.s
3	211.8	C=O	-	-	-
4a	44.2	CH_2	2.25	1	o.s
4b			2.20	1	o.s
5	42.8	CH	2.21	1	o.s
6a	29.7	CH_2	1.82	1	o.s
6b			1.22	1	o.s
7	117.0	CH	5.17	1	o.s
8	139.4	C	-	-	-
9	48.8	CH	1.73	1	o.s
10	34.4	C	-	-	-
11a	21.7	CH_2	1.61	1	o.s
11b			1.53	1	o.s
12a	39.3	CH_2	1.46	1	o.s
12b			1.24	1	o.s
13	43.2	C	-	-	-
14	55.0	CH	1.82	1	o.s
15a	22.9	CH_2	1.47	1	o.s
15b			1.40	1	o.s
16a	28.1	CH_2	1.83	1	o.s
16b			1.23	1	o.s
17	55.9	CH	1.24	1	o.s
18	12.1	CH_3	0.55	3	s
19	12.4	CH_3	0.99	3	s
20	40.5	CH	2.01	1	o.s
21	21.1	CH_3	1.00	3	d (J=7.0)
22	135.5	CH	5.17	1	o.s
23	131.9	CH	5.17	1	o.s
24	42.8	CH	1.84	1	o.s
25	33.1	CH	1.48	1	o.s
26	19.6	CH_3	0.81	3	d (J=7.0)
27	19.9	CH_3	0.80	3	d (J=7.5)
28	17.6	CH_3	0.89	3	d (J=7.0)

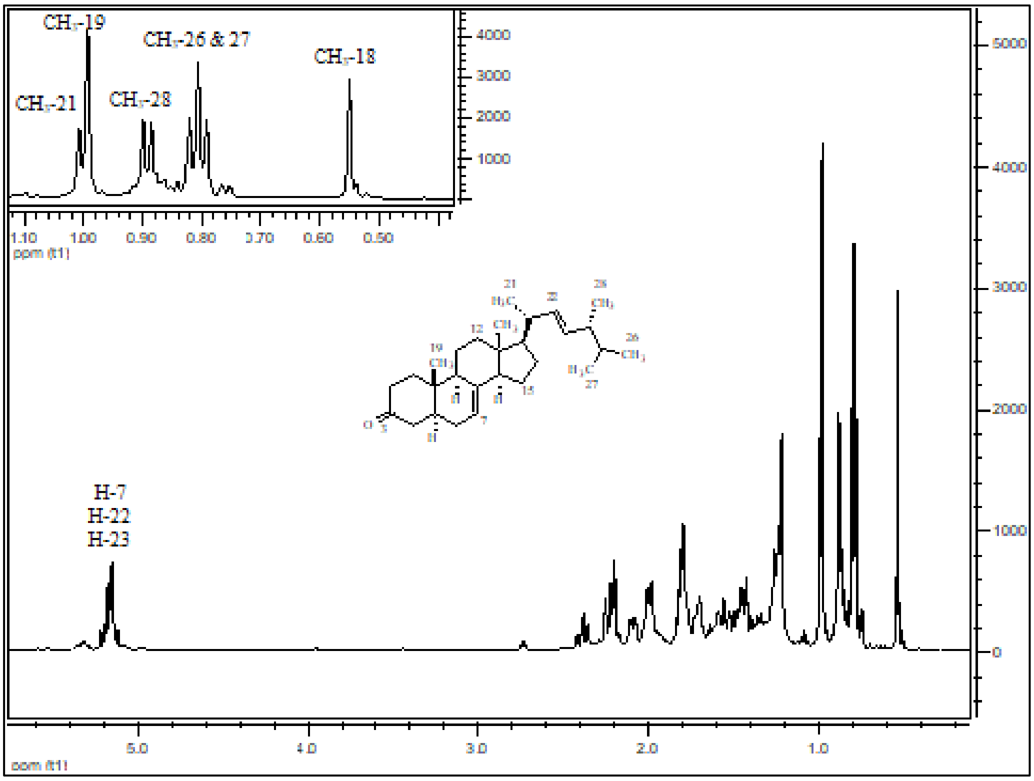

Figure S6. ^{1}H-NMR spectrum of compound **2** (CD_3OD, 500MHz)

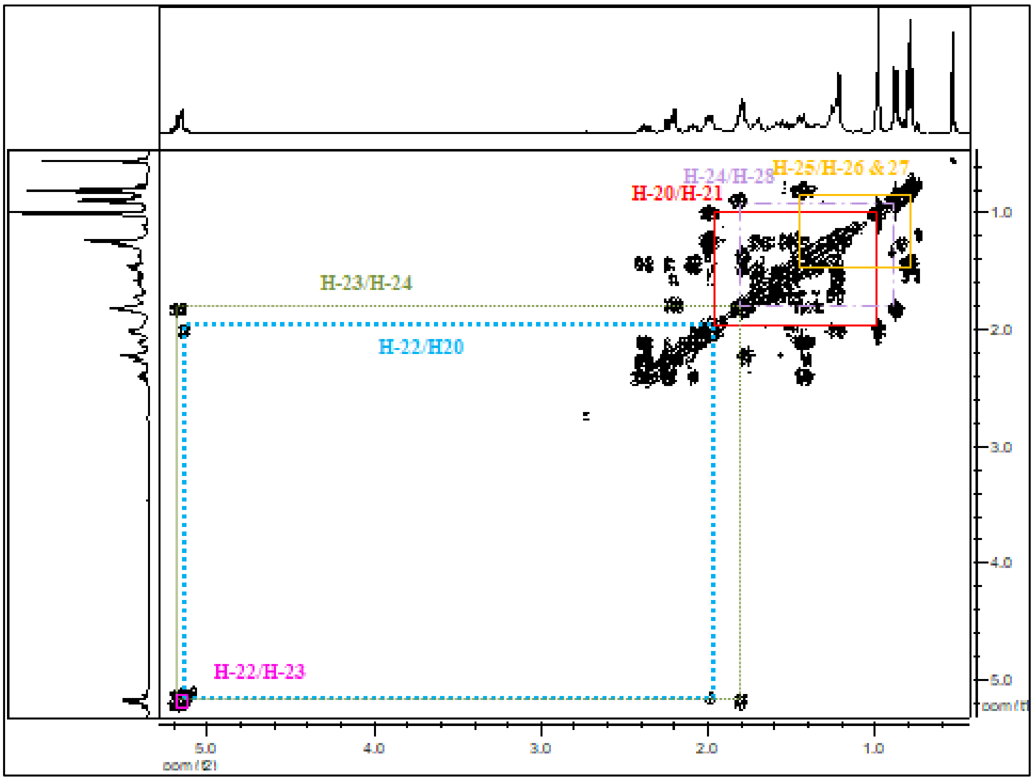

Figure S7. gDQCOSY spectrum of compound **2** (CD_3OD, 500MHz)

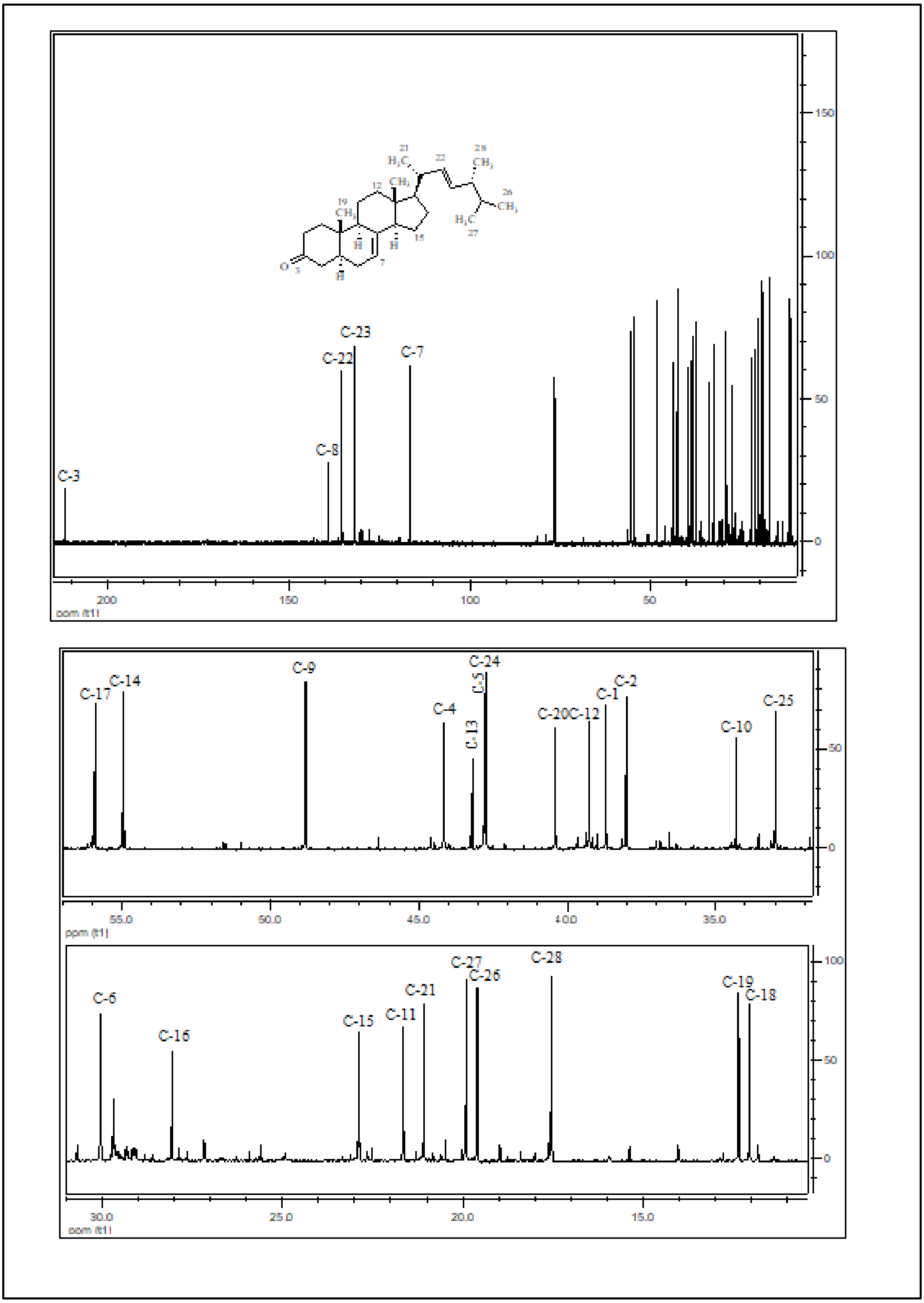

Figure S8. ^{13}C-NMR spectrum of compound **2** (CD_3OH, 125MHz)

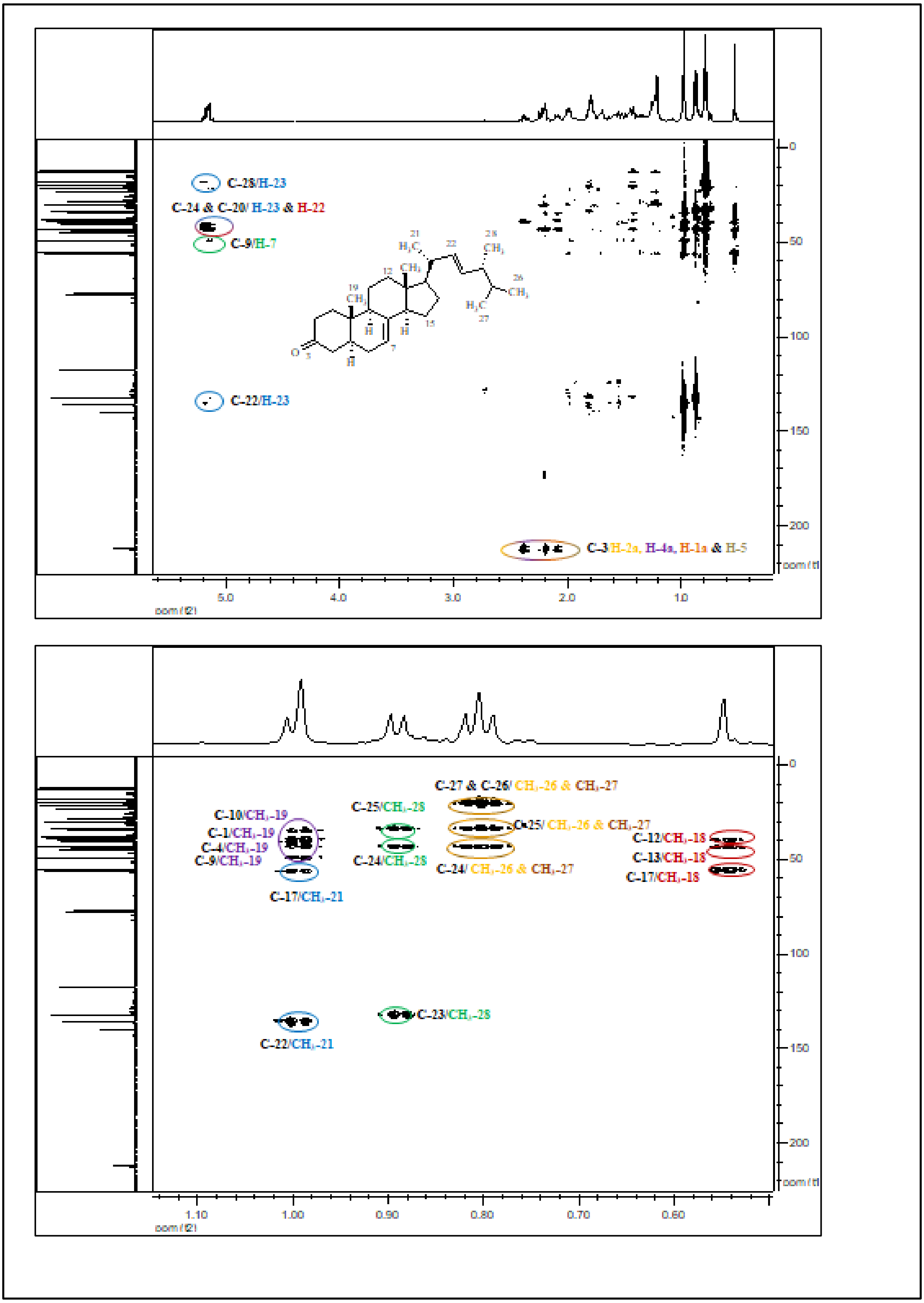

Figure S9. gHMBCAD spectrum of compound **2** (CD_3OD, 500MHz)

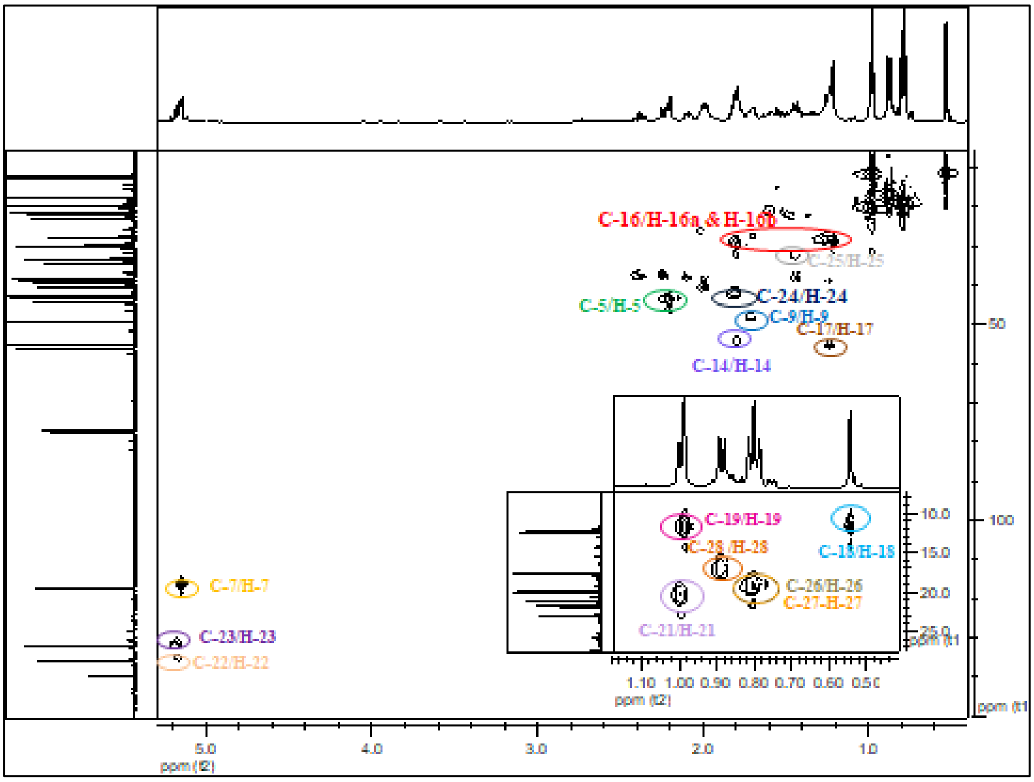

Figure S10. gHSQCAD spectrum of compound **2** (CD_3OD, 500MHz)

Table S3. NMR spectroscopic data of compound **3** ($CDCl_3$, 500MHz/125MHz)

No	δC	Type C	δH	H	Multiplicity J (Hz)
1a	37.1	CH_2		1	o.s
1b				1	o.s
2a	29.7	CH_2	1.80	1	o.s
2b			1.28	1	o.s
3	71.0	CH-OH	3.60	1	m
4a	38.0	CH_2	1.71	1	o.s
4b			1.38	1	
5	40.3	CH	2.00	1	o.s
6a	31.5	CH_2		1	o.s
6b				1	o.s
7	117.4	CH	5.16	1	o.s
8	139.5	C	-	-	-
9	49.4	CH	1.64	1	o.s
10	34.2	C	-	-	-
11a	21.1	CH_2	1.64	1	o.s
11b			1.55	1	o.s
12a	39.4	CH_2	2.01	1	o.s
12b			1.26	1	o.s
13	43.3	C	-	-	-
14	55.1	CH	1.82	1	o.s
15a	22.9	CH_2	1.57	1	o.s
15b			1.50	1	o.s
16a	28.1	CH_2	1.75	1	o.s
16b			1.24	1	o.s
17	55.9	CH	1.24	1	o.s
18	12.1	CH_3	0.55	3	s
19	13.0	CH_3	0.79	3	s
20	40.5	CH	2.01	1	o.s
21	21.5	CH_3	1.01	3	d (J=6.5)
22	135.6	CH	5.17	1	o.s
23	131.9	CH	5.20	1	o.s
24	42.8	CH	1.48	1	o.s
25	33.1	CH	1.80	1	o.s
26	19.6	CH_3	0.84	3	d (J=7.0)
27	19.9	CH_3	0.84	3	d (J=7.0)
28	17.6	CH_3	0.91	3	d (J=7.0)

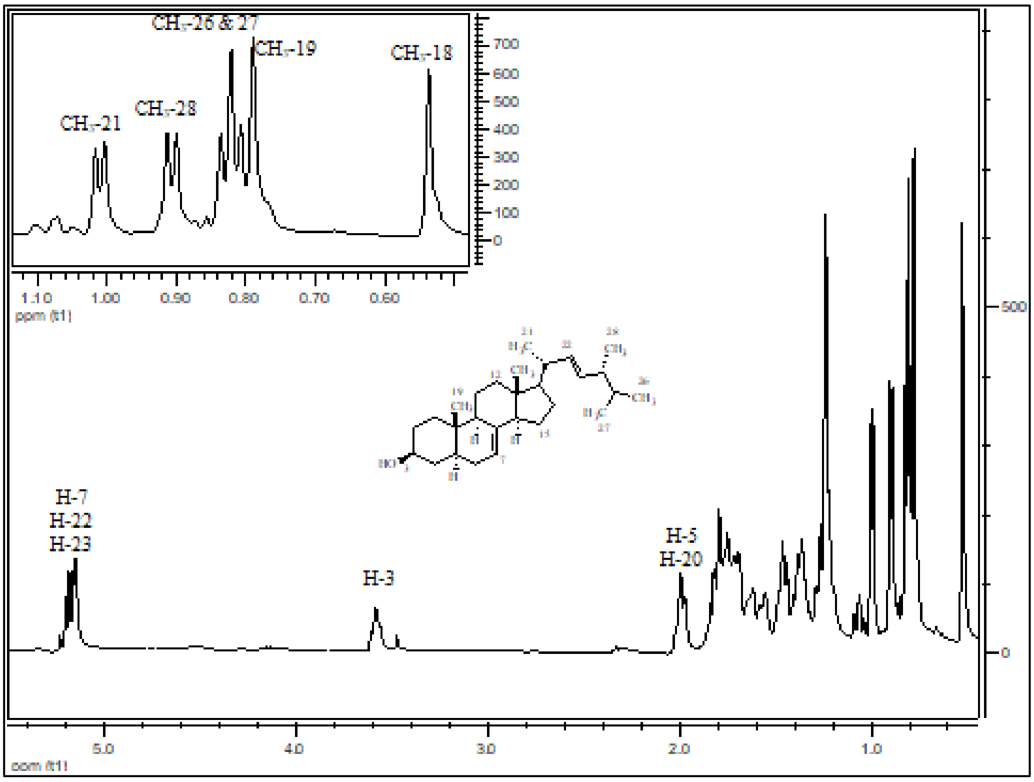

Figure S11. ^{1}H-NMR spectrum of compound **3** ($CDCl_3$, 500MHz)

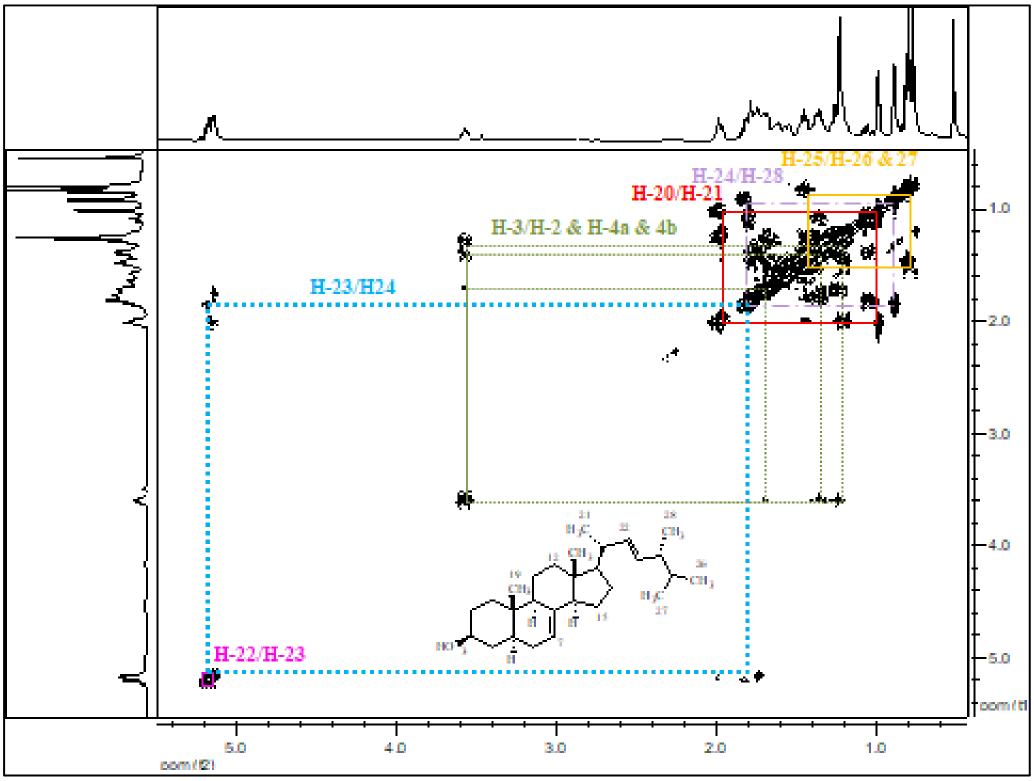

Figure S12. gDQCOSY spectrum of compound **3** ($CDCl_3$, 500MHz)

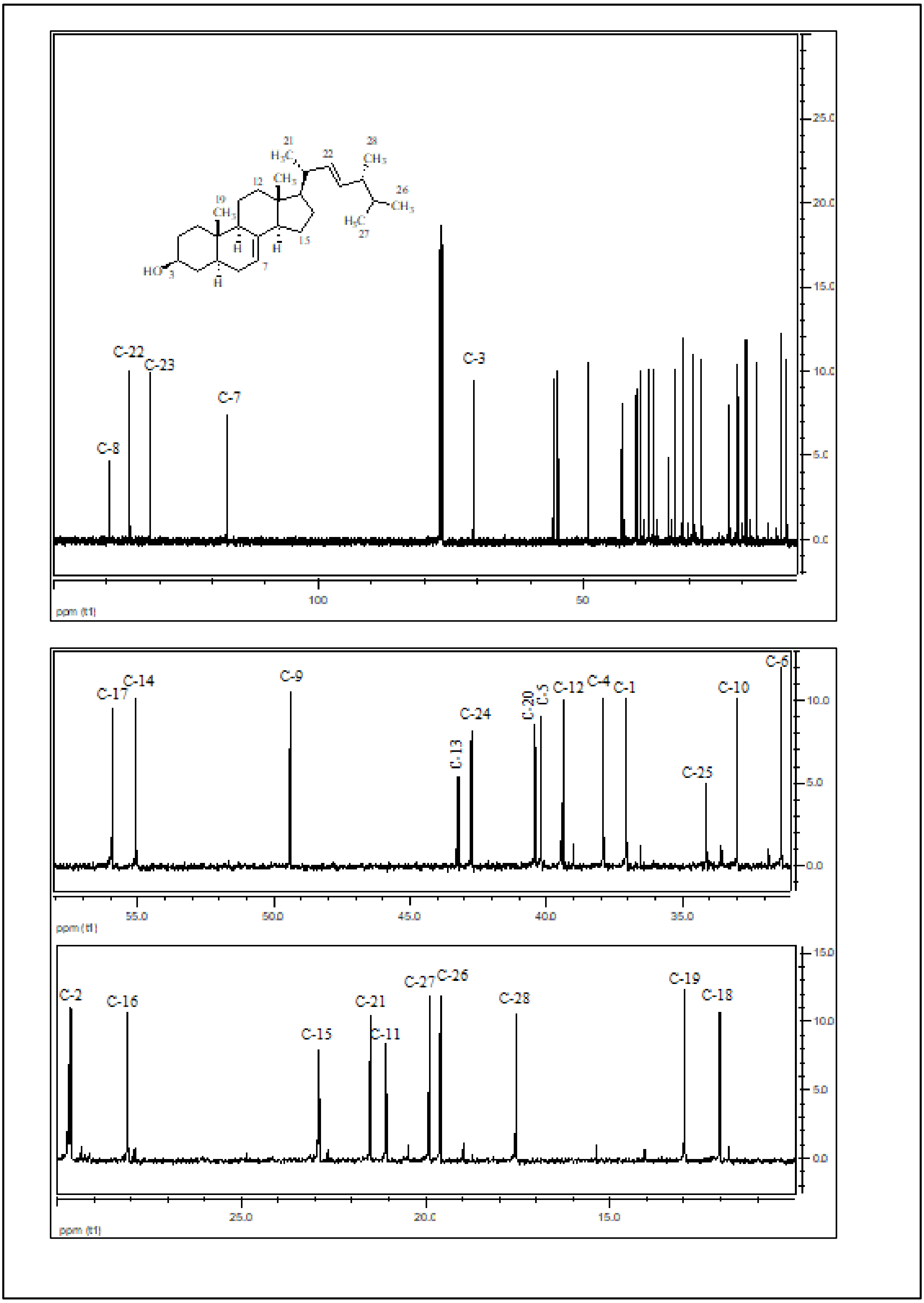

Figure S13. ^{13}C-NMR spectrum of compound **3** ($CDCl_3$, 125MHz)

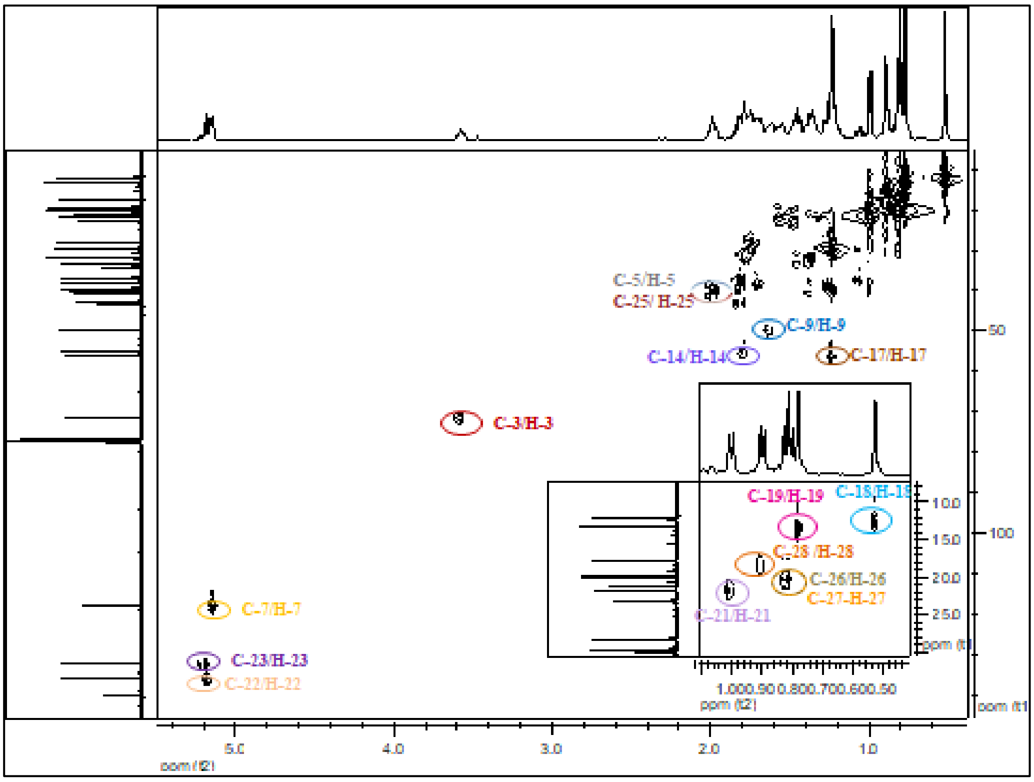

Figure S14. gHSQCAD spectrum of compound **3** ($CDCl_3$, 500MHz)

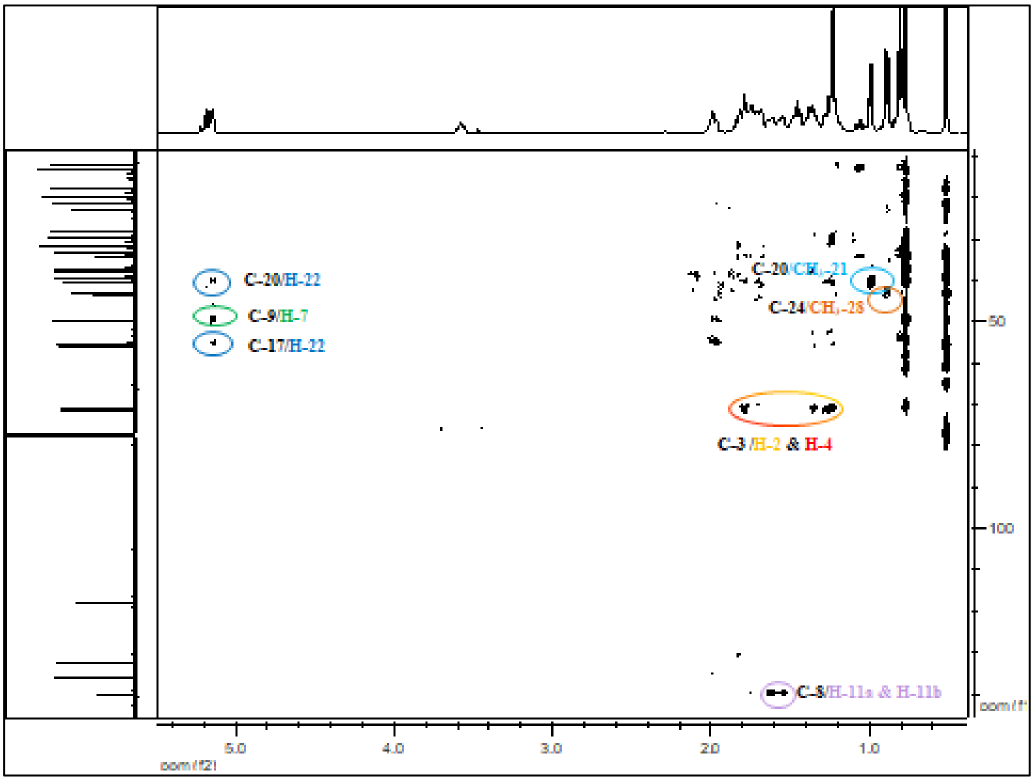

Figure S15. gHMBCAD spectrum of compound **3** ($CDCl_3$, 500MHz)

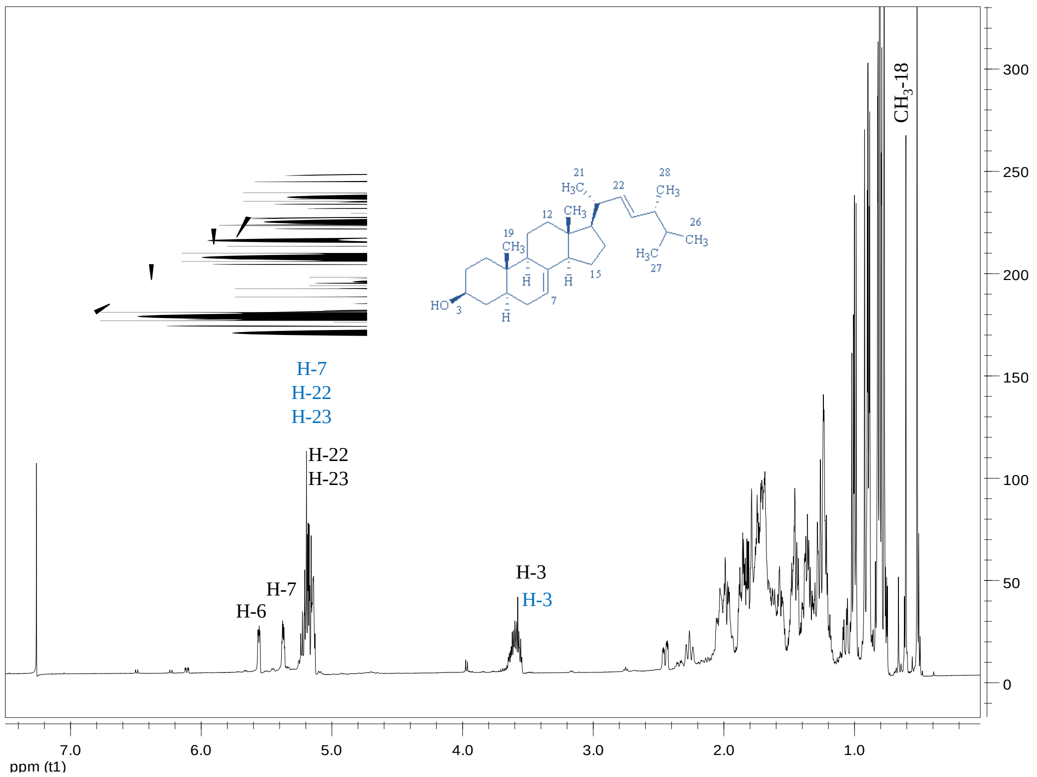

Figure S16. ^{1}H-NMR spectrum of compound **4** ($CDCl_3$, 500MHz)

Table S4. NMR spectroscopic data of compound **5** (CD_3OD, 500MHz/125MHz)

No	δ_C	Type C	δ_H	H	Multiplicity J (Hz)
1	35.5	CH_2	2.08	2	t (J=7.5)
2a	33.3	CH_2	2.48	1	ddd (J=15.5, 8.0, 7.5)
2b			2.74	1	o.s
3	217.9	C=O	-	-	-
4	45.6	C	-	-	-
5	40.5	CH	2.82	1	o.s
6a	22.9	CH_2	2.15	1	br d (J=15.0)
6b			1.91	1	t (J=13.5)
7	62.2	O-CH	4.90	1	d (J=3.5)
8	63.5	C-O	-	-	-
9	167.8	C	-	-	-
10	40.3	C	-	-	-
11	127.5	CH	6.18	1	s
12	204.2	C=O	-	-	-
13	49.9	C	-	-	-
14	64.7	C	-	-	-
15	77.7	HC-OH	4.11	1	s
16	129.7	CH	5.71	1	s
17	157.0	C	-	-	-
18	22.4	CH_3	1.66	3	s
19	27.5	CH_3	1.14	3	s
20	71.4	C	-	-	-
21	28.1	CH_3	1.47	3	s
22a	53.4	CH_2	3.00	1	o.s
22b			2.81	1	o.s
23	208.8	C=O	-	-	-
24a	45.7	CH_2	3.30	1	o.s
24b			2.63	1	dd (J=18.0, 5.0)
25	35.1	CH	2.79	1	o.s
26	179.2	O=C-OH	-	-	-
27	16.3	CH_3	1.13	3	d (J=6.0)
28	22.4	CH_3	1.10	3	s
29	23.4	CH_3	1.14	3	s
30	26.4	CH_3	1.05	3	s

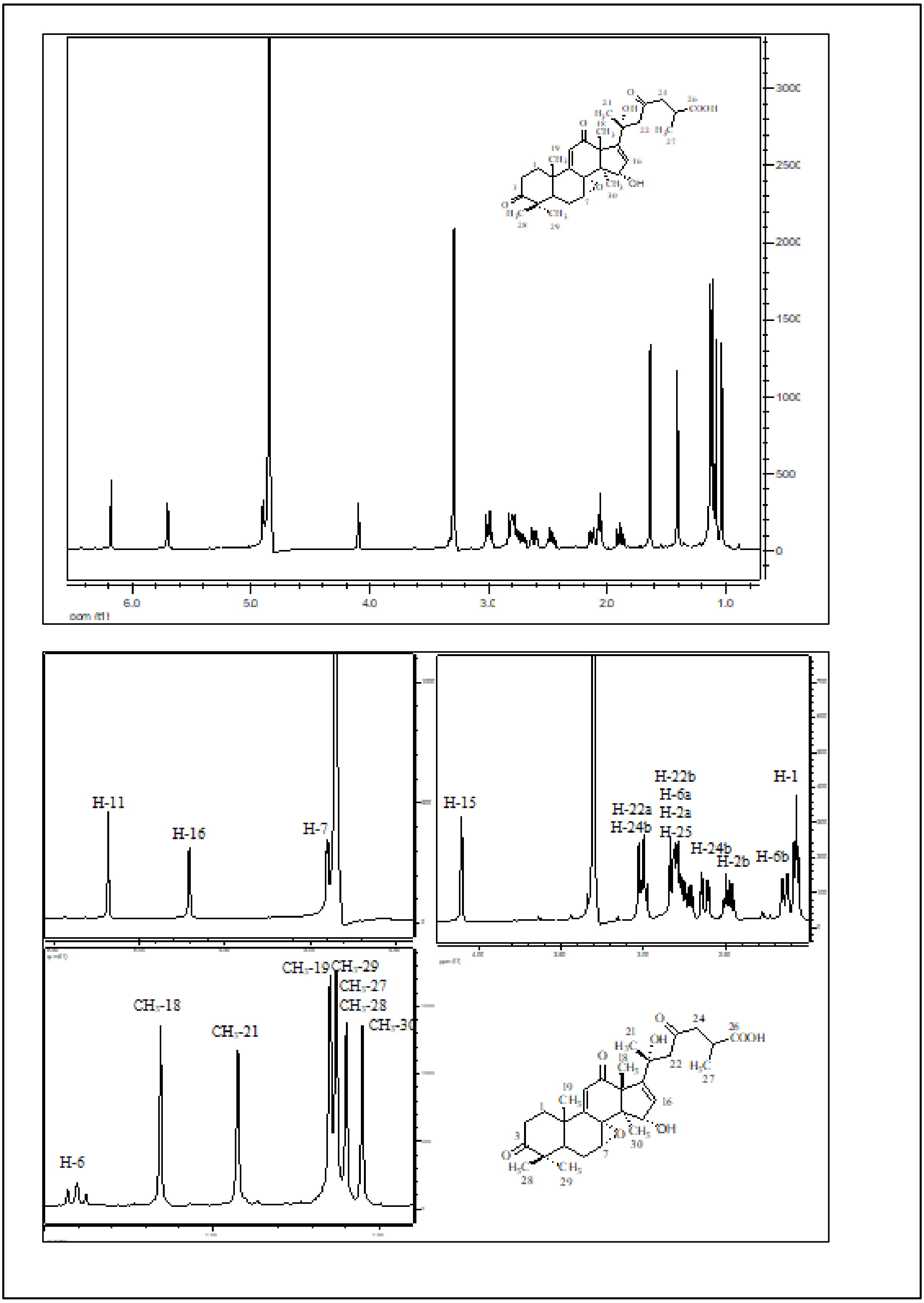

Figure S17. ^{1}H-NMR spectrum of compound **5** (CD_3OD, 500MHz)

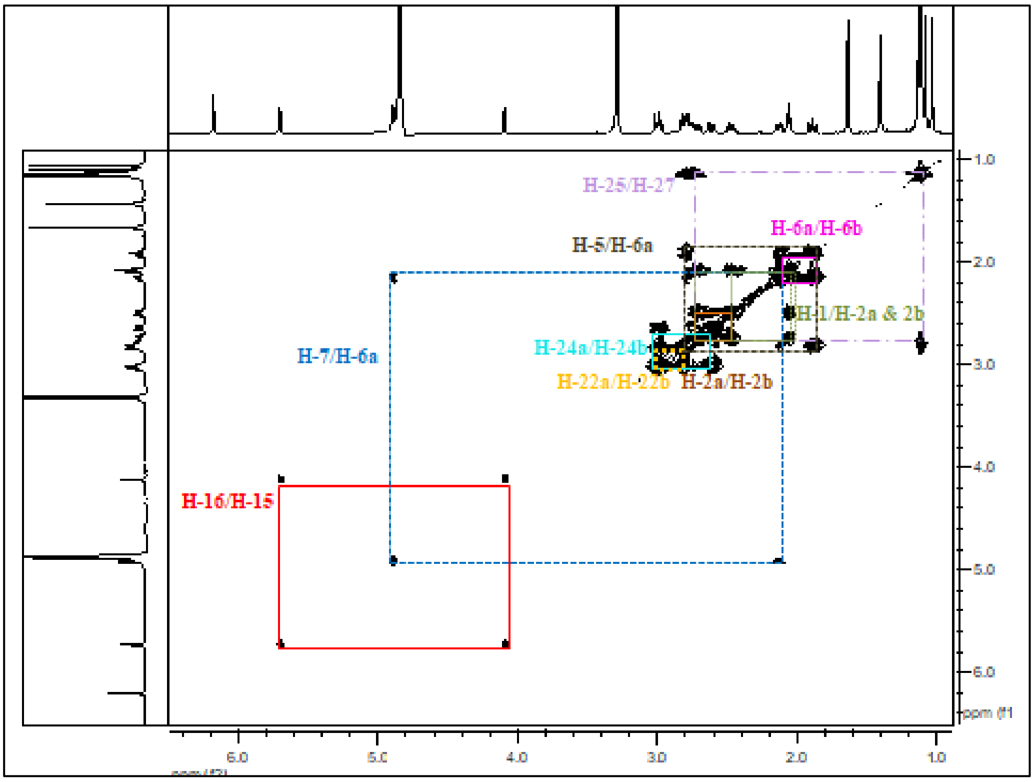

Figure S18. gDQCOSY spectrum of compound **5** (CD$_3$OD, 500MHz)

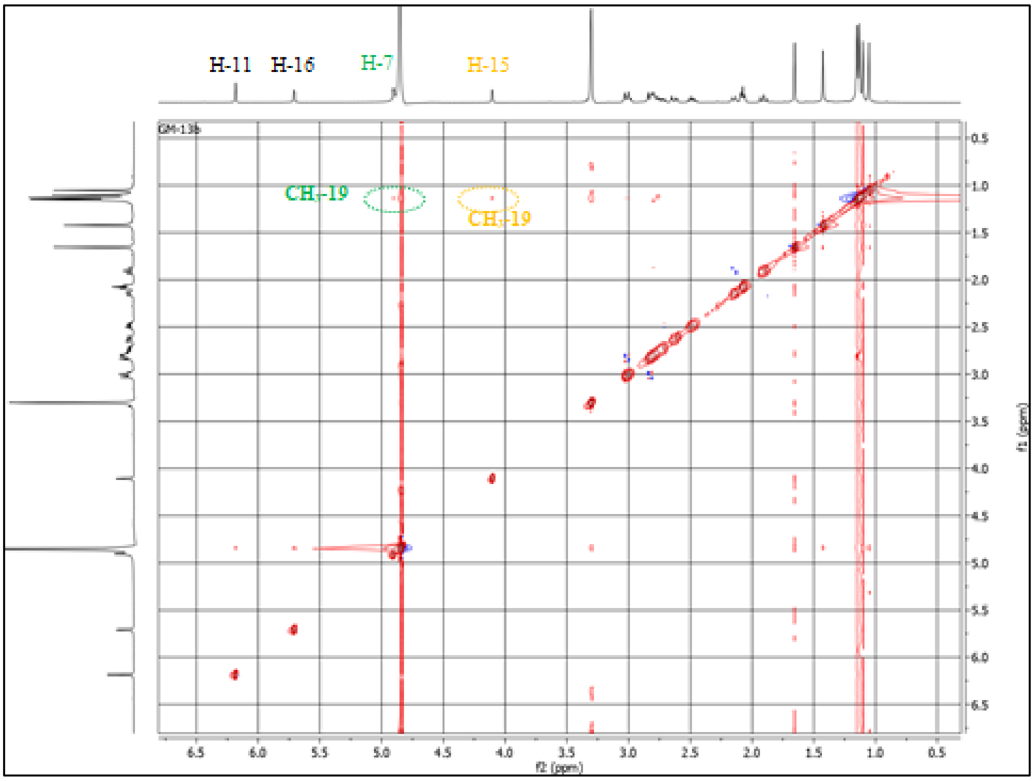

Figure S19. ROESY spectrum of compound **5** (CD$_3$OD, 500MHz)

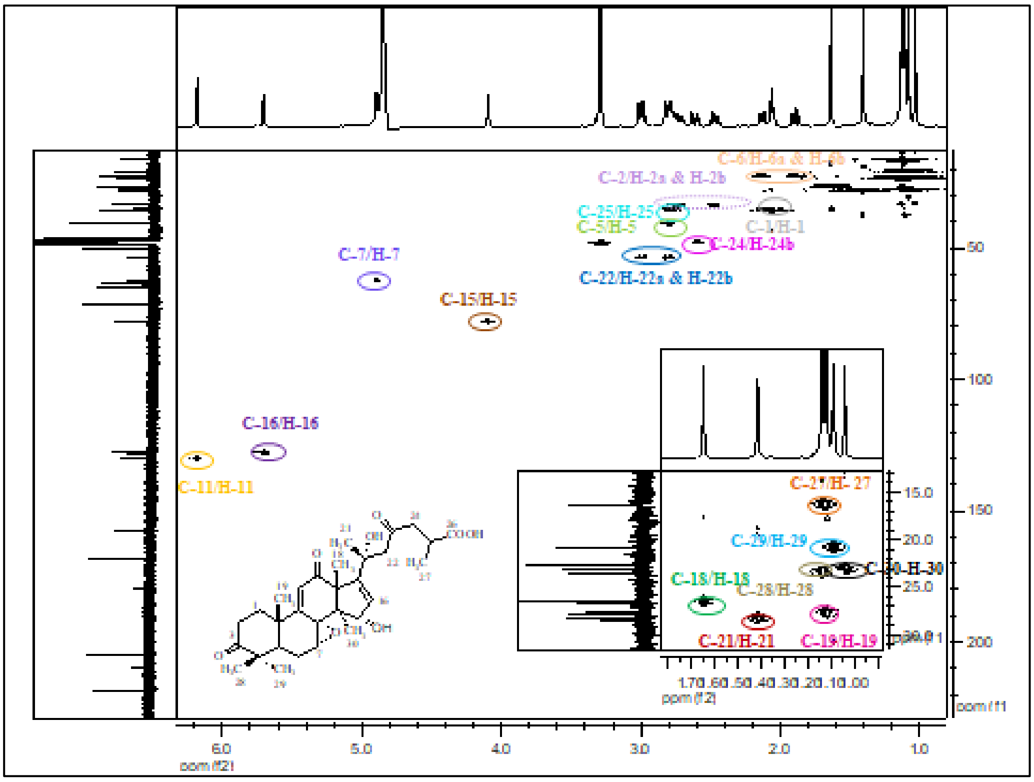

Figure S20. gHSQCAD spectrum of compound **5** (CD_3OD, 500MHz)

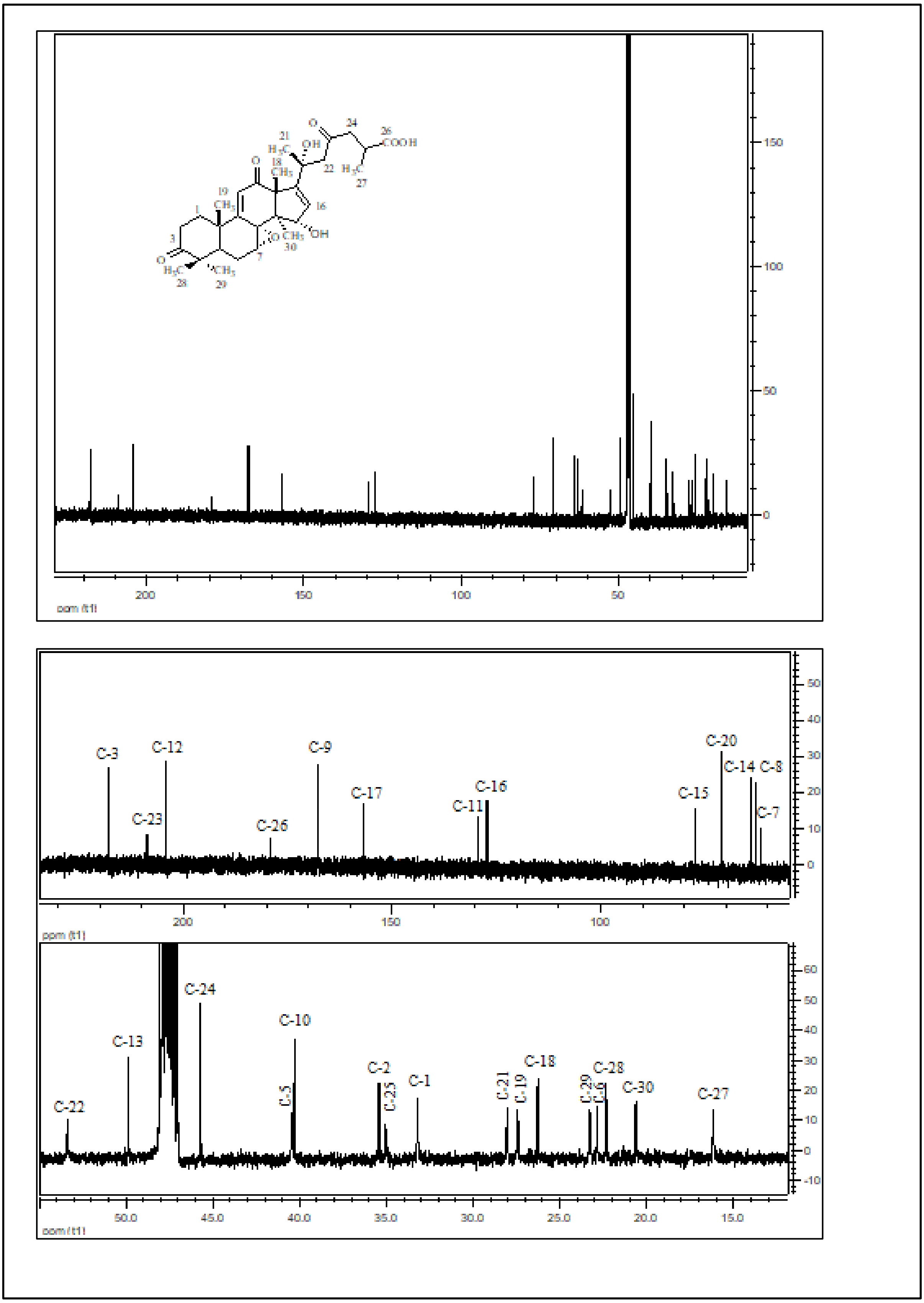

Figure S21. ^{13}C-NMR spectrum of compound **5** (CD_3OD, 125MHz)

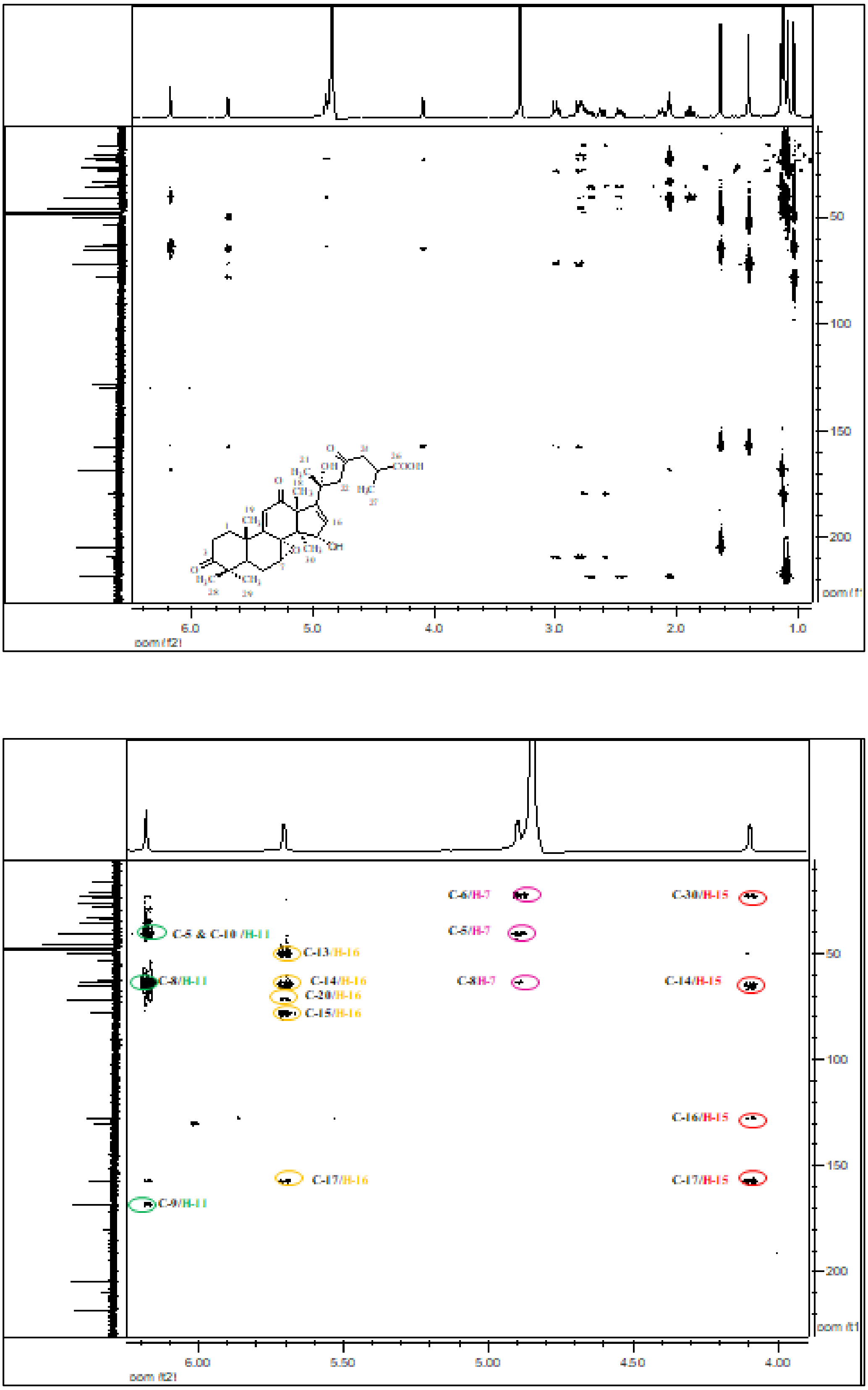

Figure S22. First part of gHMBCAD spectrum of compound **5** (CD_3OD, 125MHz) 3.8 to 6.5ppm

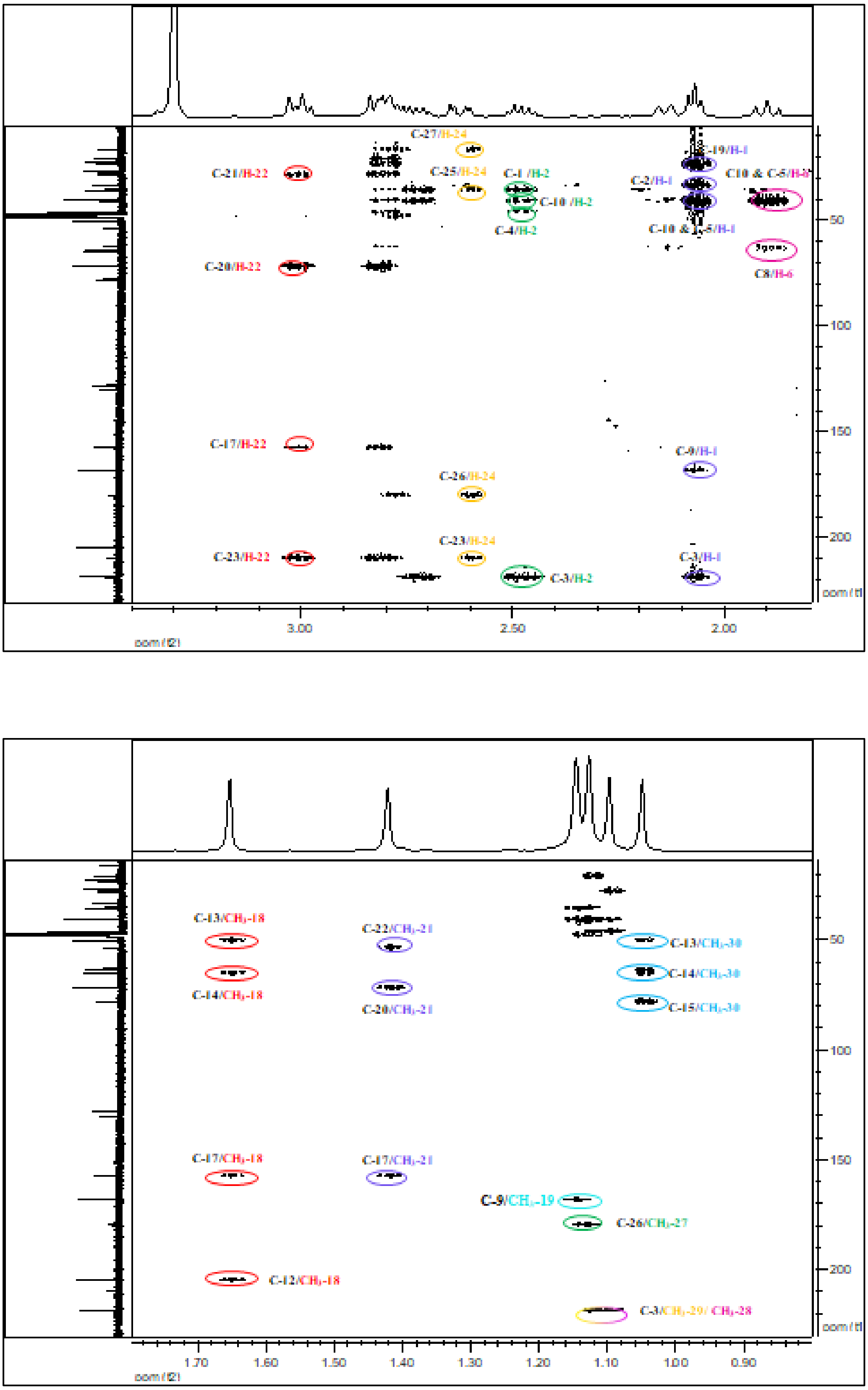

Figure S23. Second part of gHMBCAD spectrum of compound **5** from 0.8 to 3.5ppm (CD3OD, 125MHz)

Table S5. NMR spectroscopic data of compound **6** ($CDCl_3$, 500MHz)

No	δ_H	H	Multiplicity J (Hz)
1	1.95	2	m
2a	2.46	1	o.s
2b	2.67	1	o.s
3	-	-	-
4	-	-	-
5	2.74	1	d (J=13.0)
6a	2.14	1	o.s
6b	1.79	1	o.s
7	4.14	1	br s
8	-	-	-
9	-	-	o.s
10	-	-	-
11	6.04	1	s
12	-	-	-
13	-	-	-
14	-	-	-
15	4.46	1	t (J=8.0)
16a	2.46	1	o.s
16b	1.80	1	o.s
17	3.35	1	dd (J=10.5, 6.5)
18	1.00	3	s
19	1.08	3	s
20	-	-	-
21	2.18	3	s
22	6.37	1	s
23	-	-	-
24a	2.86	1	o.s
24b	2.59	1	o.s
25	2.85	1	o.s
26	-	-	-
27	1.13	3	d (J=7.5)
28	1.16	3	s
29	1.07	3	s
30	1.11	3	s

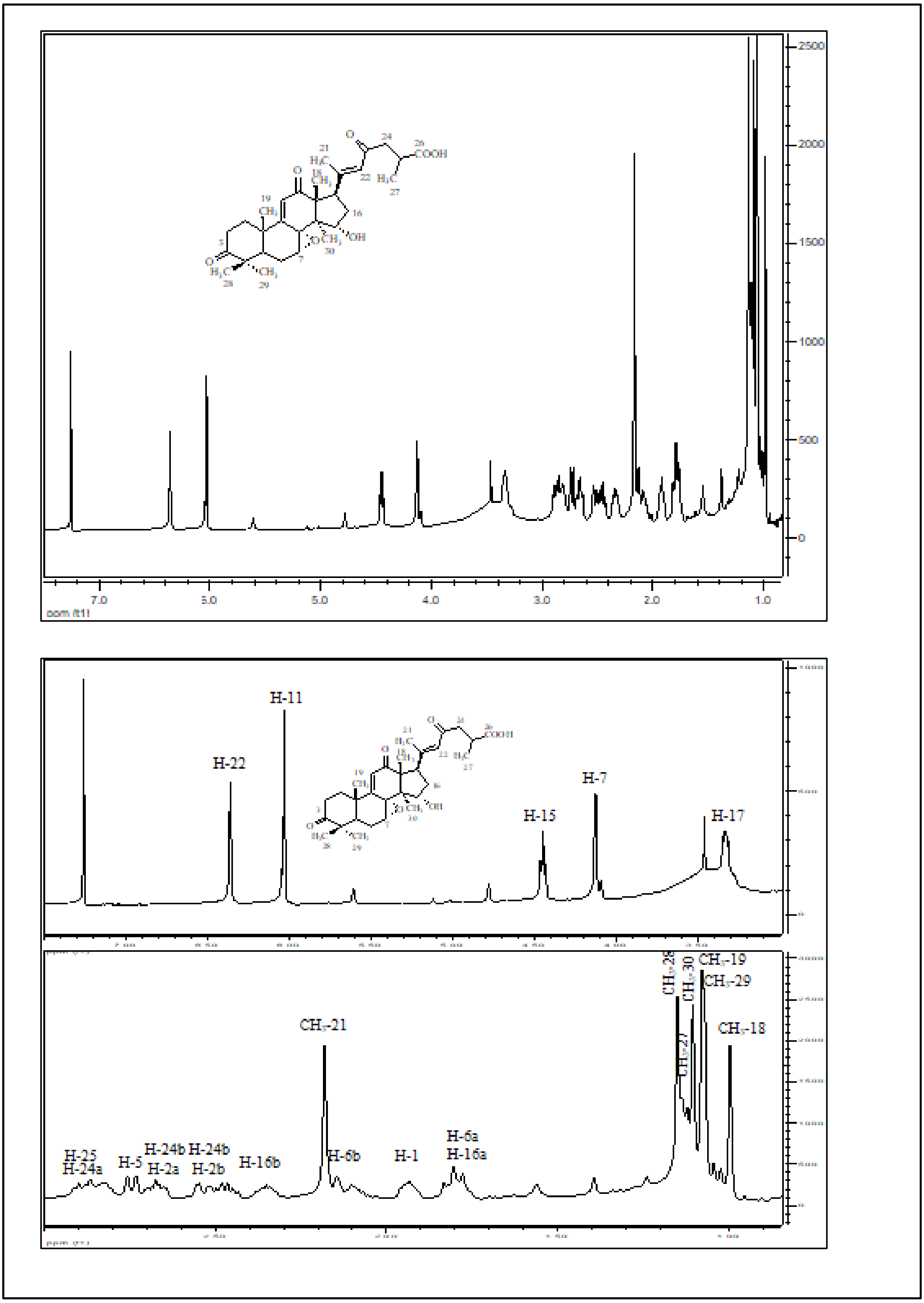

Figure S24. ^{1}H-NMR spectrum of compound **6** ($CDCl_3$, 500MHz)

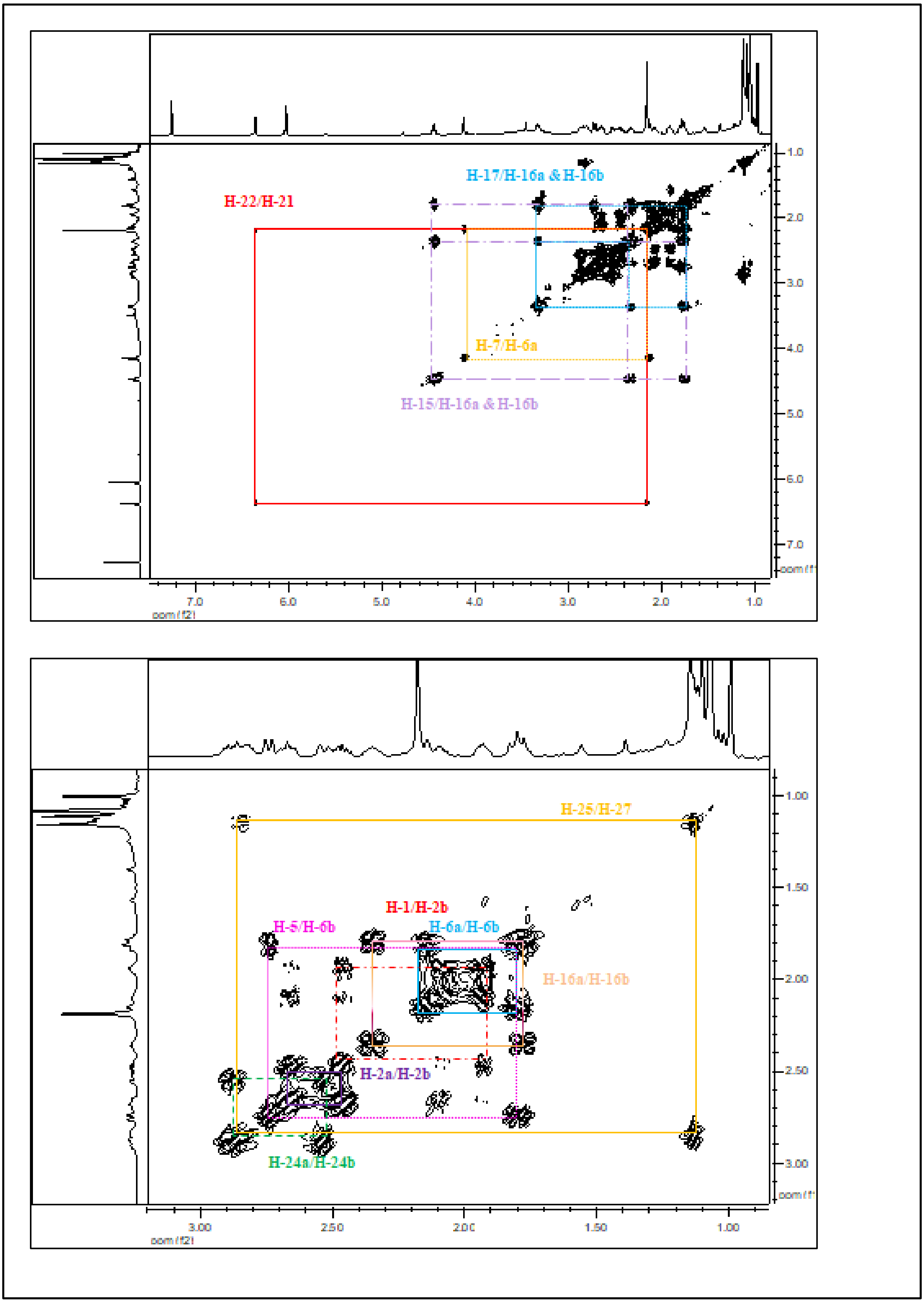

Figure S25. gDQCOSY spectrum of compound **6** ($CDCl_3$, 500MHz)
